# Design, Synthesis and Molecular Docking Studies of Some Novel Hydrazone Derivatives Based on Trimetazidine as Selective and Potent AChE Inhibitors

**DOI:** 10.1002/ddr.70382

**Published:** 2026-09-14

**Authors:** Muhammed Oğuzhan Doğan, Begüm Nurpelin Sağlık Özkan, Abdullah Burak Karaduman, Sevgi Karakuş, Yusuf Özkay, Zafer Asım Kaplancıklı, Fatih Tok

**Affiliations:** ^1^ Department of Pharmaceutical Chemistry Faculty of Pharmacy, Marmara University İstanbul Türkiye; ^2^ Department of Pharmaceutical Chemistry Institute of Health Sciences, Marmara University İstanbul Türkiye; ^3^ Department of Pharmaceutical Chemistry Faculty of Pharmacy, Anadolu University Eskişehir Türkiye; ^4^ Central Research Laboratory (MERLAB) Faculty of Pharmacy, Anadolu University Eskişehir Türkiye; ^5^ Department of Pharmaceutical Toxicology Faculty of Pharmacy, Anadolu University Eskişehir Türkiye; ^6^ Department of Pharmaceutical Chemistry Faculty of Pharmacy, Istanbul Aydin University İstanbul Türkiye

**Keywords:** AChE, donepezil, hydrazone, in silico studies, trimetazidine

## Abstract

Alzheimer's disease (AD) is a progressive neurodegenerative disease, and cholinergic dysfunction is considered one of the fundamental pathological factors of this disease. Guided by the clinically used acetylcholinesterase (AChE) inhibitor donepezil, we explored trimetazidine dihydrochloride as an accessible starting scaffold to generate donepezil‐like features and to develop new anti‐AD candidates. Accordingly, 20 novel hydrazone derivatives were designed and synthesized based on trimetazidine dihydrochloride, and their structures were confirmed by spectroscopic methods. The compounds were evaluated for in vitro inhibition of AChE and butyrylcholinesterase (BChE) using donepezil and tacrine as reference inhibitors, respectively. Several derivatives displayed pronounced AChE inhibition, whereas most compounds showed weak or negligible activity toward BChE, indicating a generally favorable AChE‐selective profile. Among the series, compounds **3p**, **3q** and **3r** exhibited selective inhibitory activity against AChE comparable to donepezil, with IC_50_ values of 0.030 ± 0.001 μM, 0.085 ± 0.003 μM and 0.034 ± 0.001 μM, respectively. To rationalize the observed activity trends, molecular docking studies were performed, suggesting that the most active ligands adopt donepezil‐like binding modes spanning the catalytic and peripheral anionic sites and are further stabilized by π–π/π–cation contacts and hydrogen‐bonding interactions within the active gorge. Overall, these findings identify trimetazidine‐derived hydrazide‐hydrazones as a promising chemotype for the development of selective AChE inhibitors and provide a basis for further optimization and advanced biological validation.

## 1 Introduction

Alzheimer's disease (AD) is a progressive neurodegenerative condition, primarily recognized for its impact on memory and cognitive abilities, though less common presentations are increasingly acknowledged (Raphael 2025). The defining pathological markers, amyloid plaques (Jamal et al. 2025) and neurofibrillary tangles (Thal et al. 2025), have been studied for over a century and remain essential for confirming a pathological diagnosis (DeTure and Dickson 2019). Efforts to advance diagnostic tools and disease‐modifying therapies are further complicated by the realization that AD is not solely a dual proteinopathy (involving amyloid and tau) but often coexists with other age‐related conditions like cerebrovascular disease and Lewy body disease (Yamali et al. 2021).

In AD, acetylcholinesterase (AChE) plays a central role in the cholinergic deficits that characterize the condition. AChE breaks down acetylcholine (ACh), and its overactivity contributes to the depletion of this critical neurotransmitter, leading to memory and cognitive impairments (Zahedi et al. 2023). AChE inhibitors, such as donepezil and rivastigmine, are key treatments aimed at increasing synaptic ACh levels (Makhaeva et al. 2015). While butyrylcholinesterase (BChE) also hydrolyzes ACh and becomes more relevant in advanced stages of AD, AChE remains the primary target for therapeutic intervention (Li et al. 2017). Thus, AChE remains the cornerstone enzyme in understanding and managing AD's cholinergic dysfunction (Behl et al. 2021).

Hydrazone structures exhibited considerable AChE inhibitory activity (Celebioglu et al. 2021; Kekeçmuhammed et al. 2023; Kucukoglu et al. 2019; Ibrahim et al. 2025). This is attributed to their ability to interact with target enzymes and receptors due to the unique ‐C = N–NH‐ pharmacophore group, which possesses both nucleophilic and electrophilic features (Ateş et al. 2021). Additionally, heterocyclic moieties are present in many bioactive compounds, and these scaffolds can be incorporated into natural products to enhance their biological efficacy, solubility, lipophilicity, polarity, and more (Jampilek 2019; Barbuceanu and Olaru 2025). The findings from various studies highlight that the *N*‐heterocyclic ring system serves as a core structure in countless synthetic compounds, demonstrating a wide spectrum of biological activities (Akhtar et al. 2017). Piperazine, a heterocyclic compound with a six‐membered ring containing two nitrogen atoms, has found diverse biological applications (Rathod et al. 2026). Specifically, numerous piperazine derivatives have been shown to exhibit neuroprotective effects and possess significant cholinesterase inhibitory activities.

**Figure 1:**
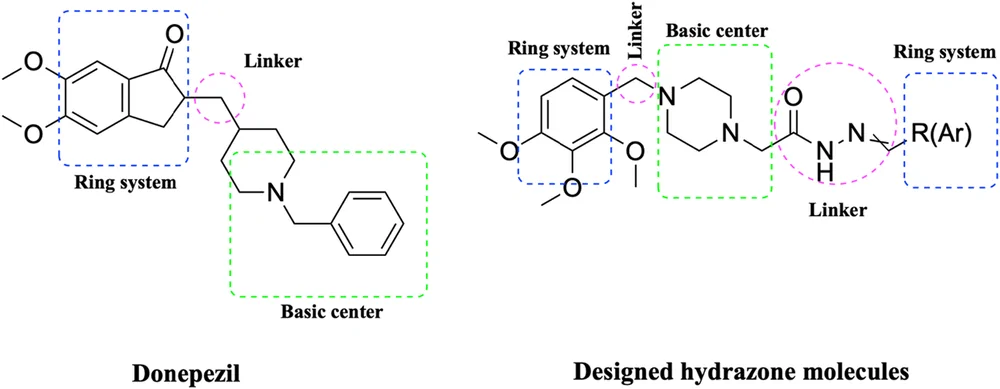
The rational design strategy of hydrazones derived from trimetazidine.

Donepezil is one of the most effective AChE inhibitors currently available on the market. It is widely used in the treatment of cognitive symptoms associated with AD (Sugimoto et al. 2000). Donepezil comprises three distinct components: an aromatic ring system (indanone), a linker (‐CH_2_‐), and a basic center (benzyl piperidine) (Almaghrabi 2025). Donepezil analogs are among the most extensively studied compounds developed as AChE inhibitors. X‐ray crystallography (PDB ID: 4EY7) and docking studies have shown that donepezil operates through a dual‐binding mechanism. The benzyl piperidine group contributes to its inhibitory effect by interacting with the catalytic active site (CAS) (CAS includes: Ser203, Glu 334, His 447, Trp 86, Tyr 130, Tyr 133, Tyr 337, Phe 338) (Colovic et al. 2013) while the indanone moiety, acting as a hydrophobic aromatic component, binds to the peripheral anionic site (PAS) (PAS includes: Tyr 72, Asp 74, Tyr 124, Tyr 341, Trp 286) (Costanzo et al. 2016; Colovic et al. 2013; van Greunen et al. 2017). Based on this information, some new hydrazone derivatives were designed from the structure of trimetazidine. The following strategy was used in designing these new molecules: (i) In the molecules designed to replace the basic center benzyl piperidine in donepezil, the piperazine ring, which is an isostere of piperidine, was inserted. (ii) Instead of the methylene group, which is the linker chain connecting the ring system and the basic center in donepezil, two linkers were preferred in the designed molecules. One of these is a methylene bridge, as in donepezil, and the other is a hydrazone group. (iii) In the compounds designed to replace the indanone group in donepezil, two substituted phenyl derivatives were observed as the ring system (Figure 1). As a result, this rational design strategy aimed to obtain new compounds that could be selective and potent AChE inhibitors similar to donepezil, based on trimetazidine.

In light of the aforementioned structural considerations, the primary aim of this study is to rationally design, synthesize, and evaluate a novel series of donepezil‐analogous compounds as potential AChE inhibitor agents. By integrating the well‐established cholinesterase‐inhibiting properties of the donepezil scaffold with bioactive hydrazone pharmacophores and N‐heterocyclic (piperazine) moieties, we aimed to develop new chemical entities with optimized interactions within the enzyme active sites. Consequently, the synthesized derivatives were systematically evaluated for their in vitro AChE and BChE inhibitory activities. Furthermore, to elucidate the structure‐activity relationships (SAR) and gain deeper mechanistic insights into the binding modes of these novel hybrids at the atomic level, comprehensive molecular docking and molecular dynamics (MD) simulations were conducted.

## 2 Results and Discussion

### 2.1 Chemistry

**Scheme 1:**
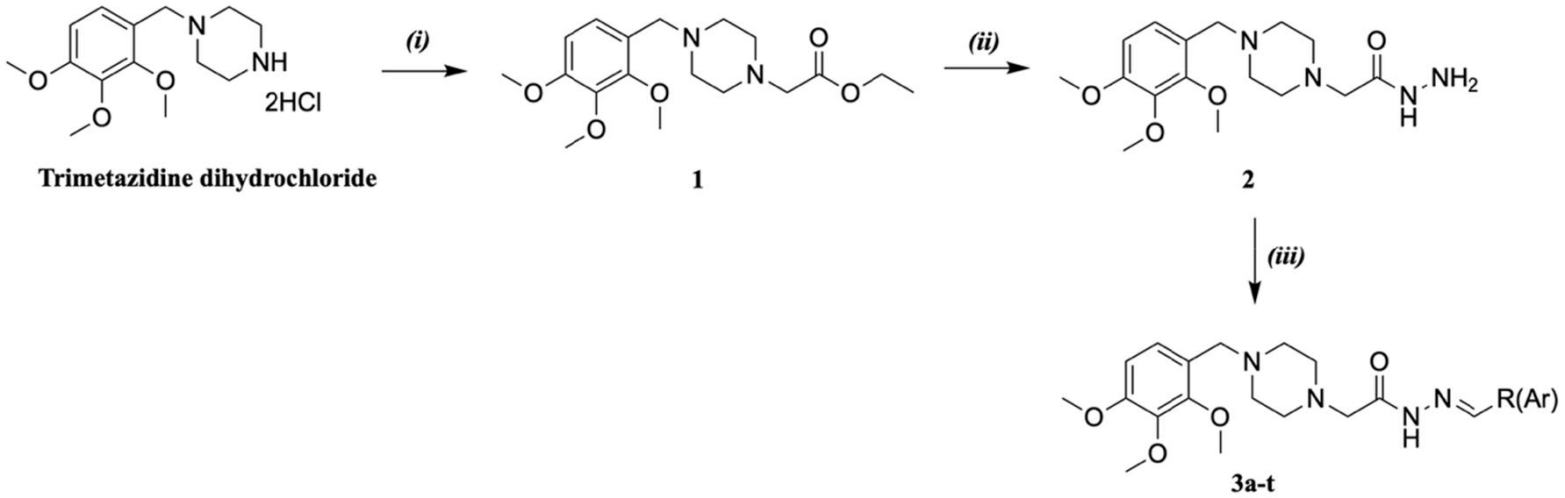
The synthetic pathway of synthesized compounds. Reagents: (i) NaHCO_3_, ethyl bromoacetate, acetone, reflux, overnight; (ii) hydrazine monohydrate, ethanol, reflux, 12 h; (iii) aryl aldehyde derivatives, ethanol, reflux, 12 h.

Target hydrazone derivatives were gained corresponding to the synthetic pathway given in Scheme 1. Initially, trimetazidine dihydrochloride is dissolved in acetone, followed by the addition of sodium bicarbonate and ethyl bromoacetate. The resulting ester compound (**1**) is then reacted with hydrazine monohydrate in an ethanol solution to obtain the hydrazide compound (**2**). The hydrazide compound (**2**) is subsequently reacted with aromatic aldehyde derivatives to obtain hydrazone derivatives (**3a**‐**t**). The structural elucidation of the compounds was carried out using FT‐IR, ^1^H‐NMR, ^13^C‐NMR, 2D‐NMR (HSQC) and elemental analysis.

In the infrared (IR) spectra of the hydrazone derivatives (**3a–t**), the N–H stretching vibrations were observed in the 3215−3488 cm^−1^ range. The carbonyl (C = O) stretching bands appeared at 1647–1716 cm^−1^, while the characteristic azomethine (C = N) stretching bands were detected at 1597−1606 cm^−1^. In addition, aromatic C = C stretching vibrations were recorded at 1492−1498 cm^−1^.

In the ^1^H‐NMR spectra, the diagnostic hydrazone N–H protons appeared as singlets at 9.59−10.36 *δ* ppm, while the azomethine (N = CH) proton resonated as a singlet in the aromatic region at 7.94−9.08 *δ* ppm. The trimethoxybenzyl OCH_3_ protons (9H) were observed at 3.75−3.93 *δ* ppm (overlapping signals), and the piperazine methylene protons appeared as multiplets at 2.39−2.93 *δ* ppm. Aromatic protons of the trimethoxybenzyl ring were recorded as doublets at 6.62−6.94 *δ* ppm. The methylene (CH_2_) protons were detected as singlets at 3.04−3.73 *δ* ppm. In addition, the phenolic –OH signals were not observed in the ^1^H‐NMR spectra of **3f**, **3n**, **3o**, and **3p**, consistent with H/D exchange (deuteration) under the measurement conditions.

In the ^13^C‐NMR spectra, the characteristic carbonyl (C = O) signals of the hydrazone derivatives were observed at 165.11–166.80 *δ* ppm. Notably, compound **3n** displayed an additional downfield resonance at 175.75 ppm, attributable to the carboxylic acid carbonyl carbon. The diagnostic azomethine (C = N) carbons of the hydrazone moiety resonated in the 144.37–149.81 *δ* ppm range. Furthermore, the piperazine ring carbons were consistently detected at approximately 51.00–54.00 *δ* ppm, in agreement with typical N‐CH_2_ environments.

**Figure 2:**
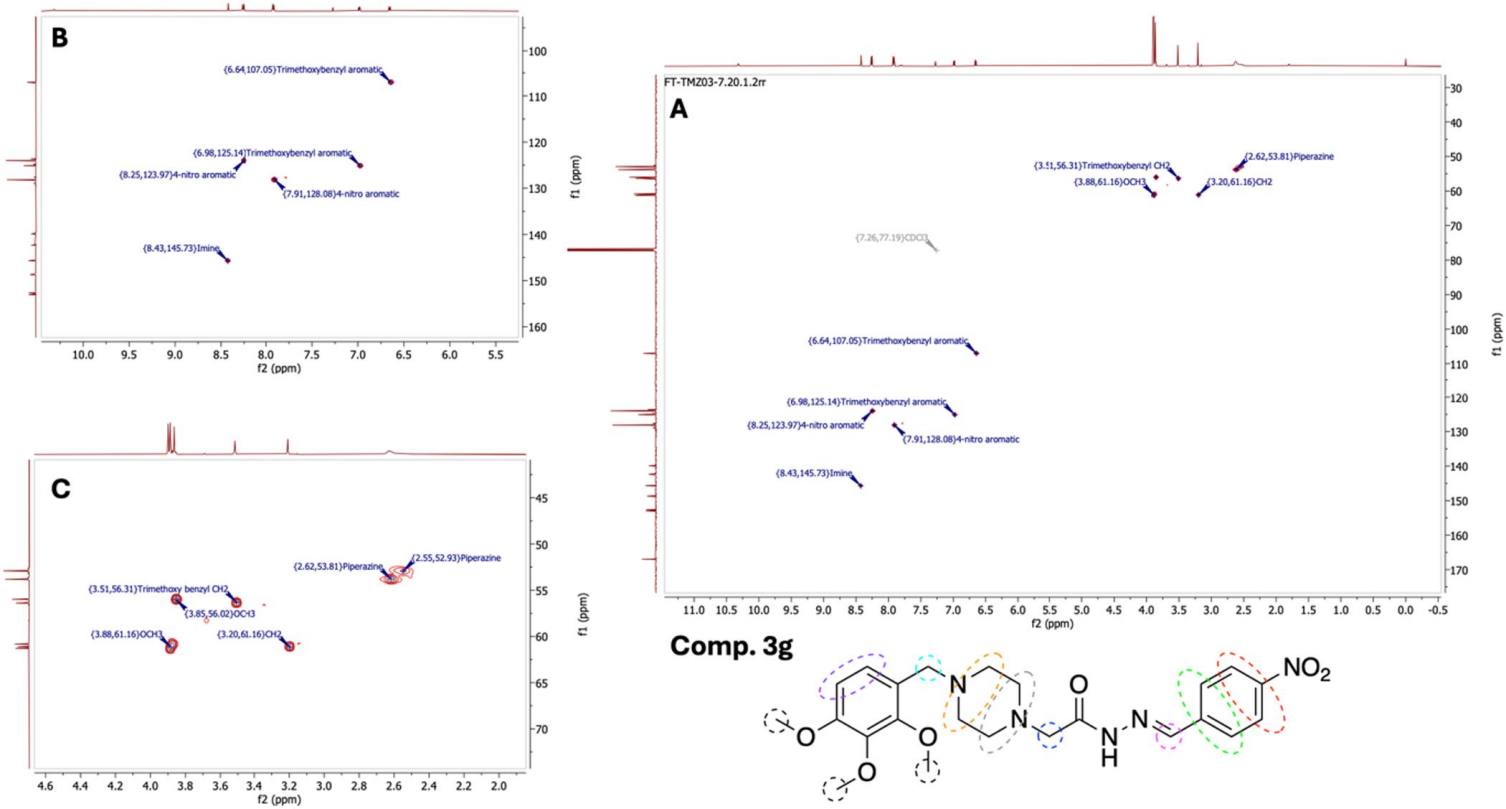
HSQC spectrum of compound **3g**: (A) full spectrum, (B) expanded aliphatic region, and (C) expanded aromatic region.

In this study, the heteronuclear single quantum coherence (HSQC) experiment was employed to assign the ^1^H–^13^C one‐bond correlations and thereby identify the carbon atoms directly bonded to the corresponding protons. In the HSQC spectrum of compound **3g**, the imine functionality was clearly evidenced by the correlation of the HC = N proton at 8.43 *δ* ppm with the C = N carbon at 145.73 *δ* ppm. As expected from the strong electron‐withdrawing character of the nitro substituent, signals belonging to the 4‐nitrophenyl ring appeared at relatively downfield positions compared with those of the trimethoxybenzyl moiety. Accordingly, aromatic carbons of the 4‐nitrophenyl ring were observed at 123.97 and 128.08 *δ* ppm, while the corresponding aromatic protons resonated at 8.25 and 7.91 *δ* ppm, respectively. For the trimethoxybenzyl fragment, the methylene group (CH_2_) attached to the aromatic ring was assigned based on its HSQC cross‐peak at 56.31 ppm/3.51 *δ* ppm. In addition, the methylene group adjacent to the carbonyl functionality showed a correlation at 61.16 ppm/3.20 *δ* ppm, consistent with deshielding induced by the nearby carbonyl group. Signals attributable to the piperazine ring were detected at 52.93 and 53.81 *δ* ppm, with the associated protons resonating at 2.55 and 2.62 *δ* ppm, respectively, in agreement with typical aliphatic N‐CH_2_ environments. Finally, the methoxy substituents of the trimethoxy group were observed within 56.02–61.16 *δ* ppm and 3.85–3.88 *δ* ppm in the HSQC spectrum, supporting their assignment to O–CH_3_ carbons and protons (Figure 2).

### 2.2 In Vitro Enzyme Studies

The trimetazidine‐derived hydrazone series (**3a–3t**) was screened for inhibitory activity against AChE and BChE according to previously described Ellman's spectrophotometric method (Acar Cevik et al. 2019; Ellman et al. 1961; Hussein et al. 2018). The resulting IC_50_ values are summarized in Table 1. Overall, the series displayed a clear selectivity for AChE over BChE, with most compounds showing negligible BChE inhibition (IC_50_ typically > 100 µM or >10 µM) while several analogues reached submicromolar to low‐nanomolar AChE potency.

**Table 1:** The IC_50_ values (μM) of compounds against AChE and BChE enzymes.^a^

Compounds	R(Ar)	AChE IC_50_ (µM)	BChE IC_50_ (µM)
**3a**	Ph	»10	>100
**3b**	4‐Me–Ph	>10	>100
**3c**	4‐F–Ph	1.508 ± 0.068	>100
**3d**	4‐Cl–Ph	0.203 ± 0.009	>10
**3e**	4‐Br–Ph	>10	>100
**3f**	4‐OH–Ph	0.105 ± 0.004	>10
**3g**	4‐NO_2_–Ph	0.184 ± 0.008	>10
**3h**	4‐OMe–Ph	0.125 ± 0.005	>10
**3i**	4‐SMe–Ph	>10	>100
**3j**	4‐CF_3_–Ph	>10	>100
**3k**	4‐NMe_2_–Ph	2.031 ± 0.095	>100
**3l**	4‐iPr–Ph	2.510 ± 0.114	>100
**3m**	4‐CO_2_Me–Ph	0.116 ± 0.005	>10
**3n**	4‐CO_2_H–Ph	0.139 ± 0.006	>10
**3o**	2,4‐(OH)_2_–Ph	1.063 ± 0.050	>10
**3p**	3,4‐(OH)_2_–Ph	0.030 ± 0.001	>10
**3q**	4‐OMe‐3‐NO_2_–Ph	0.085 ± 0.003	>10
**3r**	4‐OH‐3‐OMe–Ph	0.034 ± 0.001	>10
**3s**	5‐NO_2_‐thiophen‐2‐yl	>10	>100
**3t**	pyridin‐4‐yl	>10	>100
**Donepezil**		0.021 ± 0.001	—
**Tacrine**		—	0.0060 ± 0.0002

^a^ The test results were expressed as means of quartet assays.

Within the series, AChE inhibitory potency spanned a broad range, from inactive/weak members (IC_50_ > 10 µM) including **3a**, **3b**, **3e**, **3i**, **3j**, **3s**, and **3t** to moderately active compounds in the low‐micromolar range, namely **3c** (1.508 ± 0.068 µM), **3k** (2.031 ± 0.095 µM), **3l** (2.510 ± 0.114 µM), and **3o** (1.063 ± 0.050 µM). Notably, several derivatives emerged as highly potent AChE inhibitors (IC_50_ < 0.25 µM), including **3d** (0.203 ± 0.009 µM), **3g** (0.184 ± 0.008 µM), **3h** (0.125 ± 0.005 µM), **3m** (0.116 ± 0.005 µM), **3n** (0.139 ± 0.006 µM), **3f** (0.105 ± 0.004 µM), **3q** (0.085 ± 0.003 µM), **3r** (0.034 ± 0.001 µM), and **3p** (0.030 ± 0.001 µM). Among these, **3p** and **3r** were the most active representatives, achieving AChE inhibition in the nanomolar range (30 and 34 nM, respectively).

As expected, the reference inhibitors performed consistently under the assay conditions. Donepezil inhibited AChE with an IC_50_ value of 0.021 ± 0.001 µM, while tacrine, used as the BChE positive control, displayed an IC_50_ of 0.0060 ± 0.0002 µM. In direct comparison with the test compounds, **3p** was only ~1.4‐fold less potent than donepezil, whereas **3r** was ~1.6‐fold less potent, supporting the notion that the optimized hydrazone motif in this series can achieve donepezil‐like AChE activity under the applied experimental conditions. Importantly, despite their pronounced AChE inhibition, these top‐performing compounds remained weak against BChE (IC_50_ > 10 µM), translating into high AChE selectivity indices. Taken together, these results identify **3p** and **3r** as the primary lead candidates, while **3q**, **3f**, **3m**, **3h**, and **3n** represent sub‐0.15 µM AChE inhibitors that retain marked selectivity for AChE over BChE.

AChE‐active members of the series are characterized by aryl substitution on the hydrazone scaffold, and the most potent compounds bear the following patterns: **3p** (3,4‐(OH)_2_–Ph), **3r** (4‐OH‐3‐OMe–Ph), **3q** (4‐OMe‐3‐NO_2_–Ph), **3f** (4‐OH–Ph), **3m** (4‐CO_2_Me–Ph), **3h** (4‐OMe–Ph), and **3n** (4‐CO_2_H–Ph).

Based on structure–activity relationship (SAR) analysis, the following interpretations can be drawn:

- The parent phenyl analogue **3a** was inactive/weak against AChE (IC_50_ > 10 µM), indicating that an unsubstituted aromatic ring is insufficient for productive AChE engagement. Likewise, introducing a simple hydrophobic group at the para position (**3b**, 4‐Me–Ph) did not improve activity (still IC_50_ > 10 µM) (Bajda et al. 2013).
- Para halogenation produced mixed outcomes: the 4‐F–Ph derivative (**3c**) reached only the low‐micromolar range, whereas 4‐Cl–Ph (**3d**) improved into the submicromolar domain. Notably, the 4‐Br–Ph analogue (**3e**) remained weak, implying that excessive steric demand at the para position may be detrimental in this binding mode. A strongly electron‐withdrawing para substituent (**3g**, 4‐NO_2_–Ph) afforded submicromolar potency, whereas the para‐CF_3_ analogue (**3j**) was still weak (Sirinzade et al. 2026).
- Incorporation of a para hydroxyl group (**3f**, 4‐OH–Ph) led to a marked potency gain, consistent with an additional H‐bond donor/acceptor handle enabling stronger anchoring to residues along the catalytic gorge and/or peripheral region. The para methoxy derivative (**3h**, 4‐OMe–Ph) also displayed submicromolar activity, though slightly weaker than the phenol, supporting a preference for an oxygenated para substituent and suggesting that a free phenolic OH can provide stronger interactions than its methyl ether counterpart (Medeiros 2025).
- The most pronounced effect was observed for catechol substitution patterns. The 3,4‐dihydroxy analogue 3p emerged as the most potent inhibitor (IC_50_ = 0.030 µM), closely followed by its 4‐hydroxy‐3‐methoxy counterpart **3r** (IC_50_ = 0.034 µM). Although these compounds do not numerically surpass the highly optimized reference drug donepezil (IC_50_ = 0.021 µM), achieving such near‐equipotent nanomolar activity (approximately 1.4‐ and 1.6‐fold differences, respectively) with an unoptimized, first‐generation scaffold is a highly significant outcome. This strict positional dependence strongly indicates that the 3,4‐substitution arrangement (as seen in 3p and 3r) furnishes an optimal H‐bonding/interaction geometry compatible with the AChE gorge, whereas the 2,4‐pattern (compound 3o) likely enforces a less favorable ring orientation (Luque and Muñoz‐Torrero 2024). Consequently, rather than requiring a re‐evaluation of the design strategy, these mechanistic insights validate **3p** and **3r** as robust and highly promising lead scaffolds for future structural optimizations.
- Carbonyl‐bearing substituents supported submicromolar AChE inhibition. Both the para ester and acid derivatives were effective: **3 m** (4‐CO_2_Me–Ph; IC_50_ ≈ 0.116 µM) and **3n** (4‐CO_2_H–Ph; IC_50_ ≈ 0.139 µM). This indicates that a carbonyl‐rich polar handle is well tolerated at the para position and may contribute *via* H‐bond acceptor interactions and/or dipolar complementarity within the gorge (Luque and Muñoz‐Torrero 2024; Saxena et al. 1997).
- Heteroaryl analogues remained weak: 3s (5‐NO_2_‐thiophen‐2‐yl) and 3t (pyridin‐4‐yl) both showed IC_50_ > 10 µM. Collectively, this suggests that, within this scaffold context, a phenyl ring bearing appropriately positioned oxygenated substituents represents the preferred vector for high affinity, whereas heteroaryl replacements may disrupt aromatic stacking geometry and/or fail to project key H‐bonding features into productive enzyme regions (Luque and Muñoz‐Torrero 2024; Pandolfi et al. 2017; Sharma et al. 2025).

### 2.3 Cytotoxicity Test

The NIH/3T3 mouse embryonic fibroblast healthy cell line was employed to assess the preliminary in vitro cytotoxic profiles and safety margins of the most active derivatives, **3p** and **3r**. The NIH/3T3 line is widely recognized and standardized as a robust, non‐transformed basal cellular model for initial safety and general biocompatibility screening in early‐stage drug discovery. Its high reproducibility and broad sensitivity provide a reliable benchmark for evaluating basal cellular tolerance before moving to lineage‐specific systems.

Dose‐response curves were analyzed via non‐linear regression analysis to calculate the IC_50_ values against the computed percentage inhibition data. As presented in Table 2, compounds **3p** and **3r** exhibited cytotoxicity IC_50_ values of 29.580 ± 1.074 µM and 20.320 ± 0.912 µM, respectively. Compared to their potent AChE inhibitory activities (IC_50_ = 0.030 µM for **3p** and IC_50_ = 0.034 µM for **3r**), compounds **3p** and **3r** exerted cytotoxicity at concentrations approximately 986‐fold and 597‐fold higher than their respective therapeutic enzymatic inhibitory concentrations. These high selectivity indexes demonstrate that both compounds possess favorable safety margins and show potent AChE inhibition without exerting non‐specific basal cytotoxicity at their biologically effective concentrations.

**Table 2:** IC_50_ values of the selected compounds against the NIH/3T3 cell line.

Compounds	IC_50_ values (µM) NIH/3T3 cell line
**3p**	29.580 ± 1.074
**3r**	20.320 ± 0.912

### 2.4 In Silico Studies

#### 2.4.1 Molecular Docking Studies

**Figure 3:**
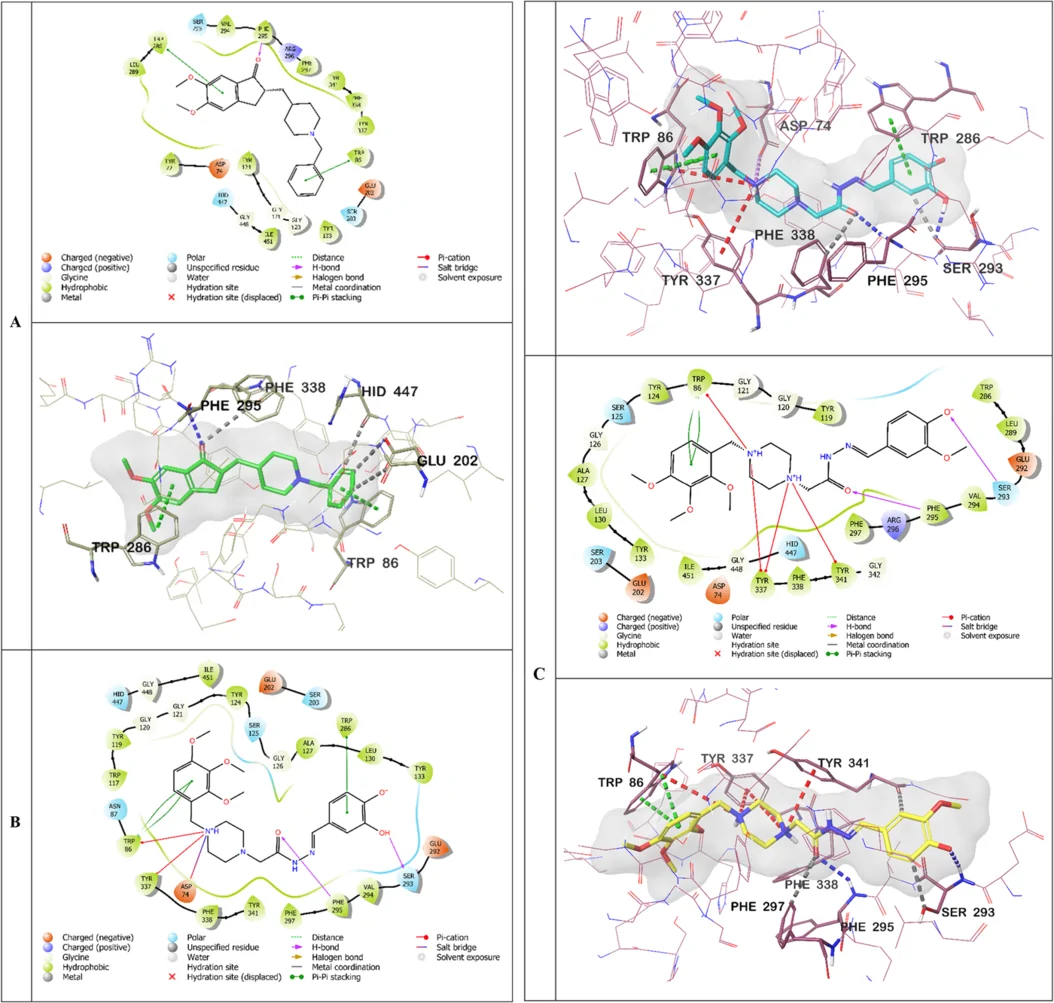
Two‐dimensional and three‐dimensional interaction profiles of donepezil (A) and compounds **3p** (B) and **3r** (C) with the active site of AChE (PDB ID: 4EY7).

To further elucidate the binding mechanisms and provide a structural rationale for the observed in vitro anticholinesterase activities, molecular docking simulations were performed on the human AChE enzyme (PDB ID: 4EY7). Based on their superior inhibitory profiles, compounds **3p** and **3r** were selected for detailed interaction analysis alongside the reference drug, donepezil. The predicted binding orientations and specific ligand‐enzyme interactions are illustrated in Figure 3.

As depicted in Figure 3A, the reference molecule donepezil exhibits a characteristic dual‐binding mode, simultaneously spanning the CAS and the PAS of AChE. The indanone moiety of donepezil is stabilized within the PAS *via* π‐π stacking interactions with Trp286 and Phe295, while its benzyl piperidine fragment is oriented towards the CAS, forming crucial π‐π interactions with Trp86. Additionally, a hydrogen bond is formed between the carbonyl oxygen of the indanone ring and the backbone amide of Phe295, further anchoring the molecule within the enzymatic gorge (Sağlık et al. 2022).

The docking results reveal that compounds **3p** and **3r** adopt a binding orientation remarkably similar to that of donepezil, effectively occupying both the CAS and PAS regions (Figure 3B,C). The aromatic scaffolds of these compounds are involved in robust π‐π stacking interactions with the indole ring of Trp286 in the PAS and Trp86 in the CAS, facilitating a stable conformation throughout the active site gorge. A significant feature of the binding profile for both **3p** and **3r** is the presence of strong π‐cation interactions (represented in red in the 3D models) between the protonated nitrogen atoms of the piperazine ring of the ligands and the aromatic residues Trp86, Tyr337, and Tyr341. These interactions are known to be pivotal for high‐affinity binding within the CAS region.

Furthermore, the stability of the enzyme–ligand complexes is reinforced by a network of hydrogen bonds. The linker regions of **3p** and **3r**, containing carbonyl and NH functionalities, form key hydrogen‐bonding interactions with Phe295 and Ser293. In certain poses, salt bridge formations (represented in pink) were also observed between the cationic centers of the ligands and the carboxylate side chain of Asp74. Analysis of the docking poses for **3p** and **3r** specifically indicates that the substitution pattern on the phenyl ring serves as a critical determinant of inhibitory activity. For these compounds, substituents at the 3rd position with hydrogen‐bonding capacity were observed to interact directly with the Ser293 residue. However, the lack of such a pronounced effect in other derivatives substituted only at the 3rd position suggests that this position alone is insufficient for optimal binding; rather, a synergistic effect between the 3rd and 4th positions appears essential. The precise role of the 4th position—specifically, how it influences the conformational orientation of the molecule or whether it contributes to direct or indirect hydrogen bonding—can be further elucidated through MD simulations (MDS). In summary, the presence of substituents capable of forming hydrogen bonds at both the 3rd and 4th positions provides a structural rationale for the prominent in vitro inhibitory potencies of compounds **3p** and **3r** identified during the molecular docking studies.

#### 2.4.2 MD Studies on AChE Enzyme

MDS were conducted for a duration of 100 ns utilizing the crystal structure of AChE (PDB ID: 4EY7) to evaluate the binding stability and dynamic behavior of compound **3p**. The simulation results revealed that the complex formed between this ligand and the 4EY7 protein remained structurally stable throughout the entire simulation period. The overall structural integrity and conformational consistency of the ligand‐enzyme complex were rigorously evaluated through root mean square deviation (RMSD) and root mean square fluctuation (RMSF) analyses, as well as the evaluation of the interaction profiles of key amino acid residues.

**Figure 4:**
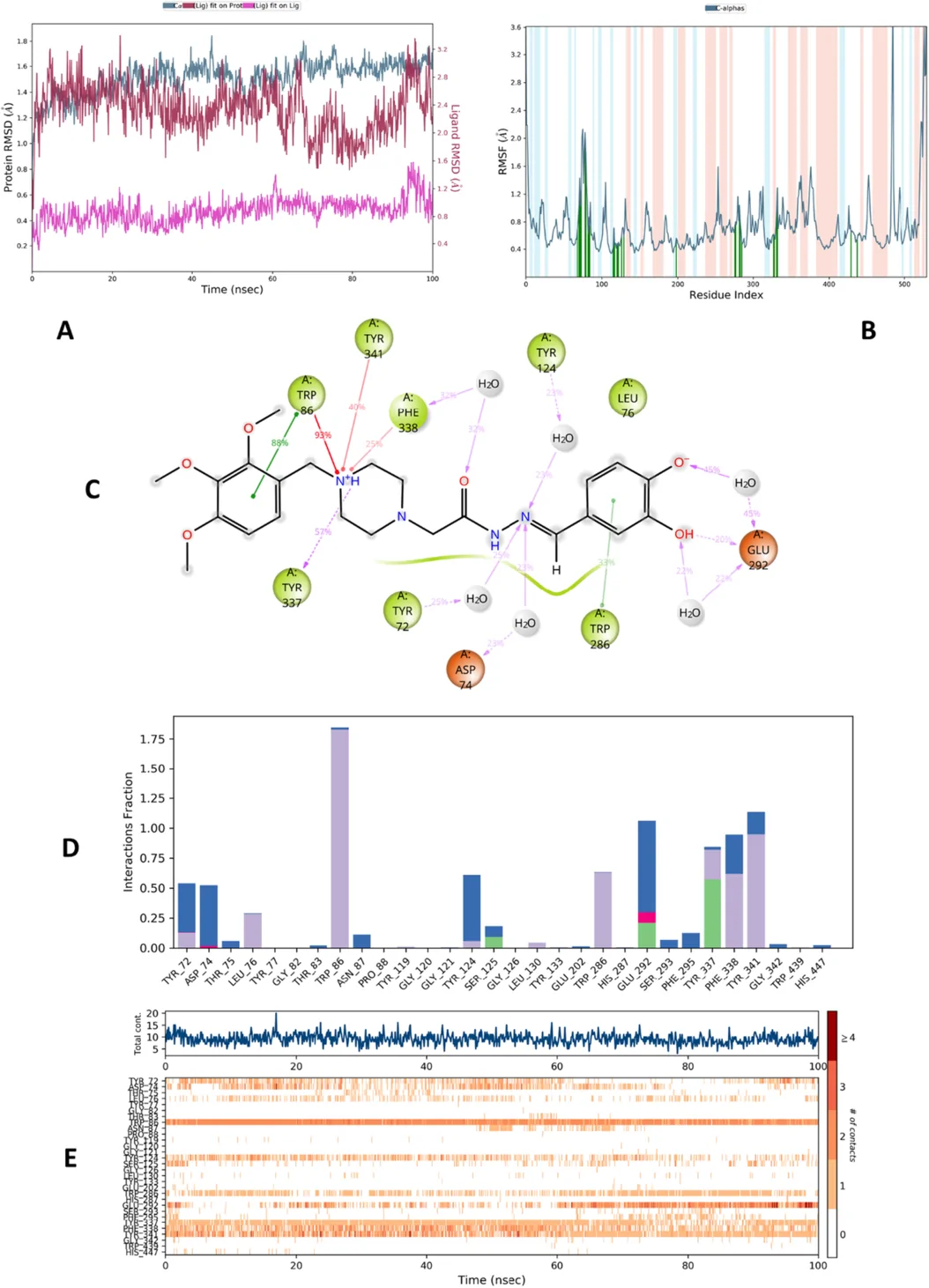
Molecular dynamic results (A−E) of compound **3p‐4EY7** complex.

The global stability of the complex was confirmed by tracking the backbone and ligand RMSD trajectories (Figure 4A). Following a rapid initial relaxation phase (~10 ns), the protein C‐alpha backbone converged to a steady equilibrium plateau with a mean RMSD of 1.6 Å, well below the conventional stability threshold (<3.0 Å). The internal ligand conformation (Fit on Ligand) remained exceptionally rigid and stable, fluctuating narrowly between 0.8 and 1.2 Å, demonstrating that compound **3p** preserved its active bioactive conformation without internal strain. The ligand‐to‐protein RMSD (Fit on Protein) stabilized around 2.8 Å after mild conformational adaptation within the binding gorge. Furthermore, local flexibility was quantified via residue‐level RMSF profiling (Figure 4B, Table 3). Key active site and catalytic gorge residues exhibited remarkably low fluctuation values, such as Tyr72 (0.70 Å), Asp74 (1.02 Å), Thr75 (1.43 Å), Leu76 (1.03 Å), Tyr77 (1.26 Å), Gly82 (2.08 Å), Thr83 (1.77 Å), Trp86 (0.63 Å), Asn87 (0.87 Å), Pro88 (0.74 Å), Tyr119 (0.42 Å), Gly120 (0.71 Å), Gly121 (0.46 Å), Tyr124 (0.42 Å), Ser125 (0.45 Å), Gly126 (0.50 Å), Leu130 (0.57 Å), Tyr133 (0.59 Å), Glu202 (0.56 Å), Trp286 (0.75 Å), His287 (0.80 Å), Glu292 (1.04 Å), Ser293 (0.80 Å), Phe295 (0.50 Å), Tyr337 (0.61 Å), Phe338 (0.59 Å), Tyr341 (0.96 Å), Gly342 (1.29 Å), Trp439 (0.63 Å), His447 (0.54 Å). These suppressed RMSF values quantitatively substantiate that binding of **3p** effectively rigidifies the catalytic pocket and PAS.

**Table 3:** RMSD, RMSF and Rg parameters (Å) for compound 3p on AChE enzyme.

Complex	RMSD	Rg	RMSF
**3p‐4EY7**	1.6 Å	5.5−6 Å	Tyr72 (0.70 Å), Asp74 (1.02 Å), Thr75 (1.43 Å), Leu76 (1.03 Å), Tyr77 (1.26 Å), Gly82 (2.08 Å), Thr83 (1.77 Å), Trp86 (0.63 Å), Asn87 (0.87 Å), Pro88 (0.74 Å), Tyr119 (0.42 Å), Gly120 (0.71 Å), Gly121 (0.46 Å), Tyr124 (0.42 Å), Ser125 (0.45 Å), Gly126 (0.50 Å), Leu130 (0.57 Å), Tyr133 (0.59 Å), Glu202 (0.56 Å), Trp286 (0.75 Å), His287 (0.80 Å), Glu292 (1.04 Å), Ser293 (0.80 Å), Phe295 (0.50 Å), Tyr337 (0.61 Å), Phe338 (0.59 Å), Tyr341 (0.96 Å), Gly342 (1.29 Å), Trp439 (0.63 Å), His447 (0.54 Å)

The molecular interactions governing binding affinity were quantitatively categorized into hydrogen bonds, hydrophobic contacts, ionic interactions, and water‐mediated bridges across the 100 ns trajectory (Figure 4C–E, Table 4). Compound **3p** established a robust interaction fingerprint spanning both the CAS and PAS. Quantitative contact occupancy analysis revealed outstanding persistence for key anchoring interactions:

- *Trp86:* Displayed the highest contact occupancy, engaging in a simultaneous cation‐π interaction with a 93% trajectory occupancy and a strong π‐π stacking interaction maintained for 88% of the simulation time.
- *Tyr337:* Formed a highly stable direct hydrogen bond with a 57% occupancy fraction.
- *Tyr341 and Phe338:* Contributed continuous stabilizing forces via cation‐π contacts with 40% and 25% occupancies, respectively, alongside a 32% water‐mediated hydrogen bonding bridge with Phe338.
- *Glu292:* Formed an extensive direct and water‐assisted network, featuring direct hydrogen bonding (20% occupancy) and multiple water‐mediated bridges at 45% and 22% occupancies.
- *Trp286:* Maintained consistent hydrophobic and π‐π stacking contacts with a 33% occupancy.

**Table 4:** Interacting amino acids and fractions for compound 3p on AChE enzyme.

Complex	Amino acids interacting above 20%	Interaction fractions
**3p‐4EY7**	Tyr72 (Water‐mediated H‐bond: 25%)	***Water‐mediated H‐bond (blue)***
	Asp74 (Water‐mediated H‐bond: 23%)	Tyr 72, Asp74, Thr75, Leu76, Gly82, Thr83, Trp86, Asn87, Pro88, Gly120, Gly121, Tyr124, Ser125, Gly126, Tyr133, Glu202, Trp286, His287, Glu292, Ser293, Phe295, Tyr337, Phe338, Tyr341, Gly342, His447
	Trp86 (π‐π interaction: 88%)	
	Trp86 (cation‐π interaction: 93%)	
	Tyr124 (Water‐mediated H‐bond: 23%)	
	Trp286 (π‐π interaction: 33%)	***H‐bond (green)***
	Glu292 (H‐ bond: 20%)	Ser125, Glu292, Tyr337, Tyr341
	Glu292 (Water‐mediated H‐bond: 45% and 22%)	
	Tyr337 (H‐ bond: 57%)	***Ionic interaction (pink)***
	Phe338 (Water‐mediated H‐bond: 32%)	Tyr72, Asp74, Glu292
	Phe338 (cation‐π interaction: 25%)	***Hydrophobic interaction (purple)***
	Tyr341 (cation‐π interaction: 40%)	Tyr72, Leu76, Trp86, Tyr119, Tyr124, Leu130, Trp286, Tyr337, Phe338, Tyr341, Trp439

Figure 4D displays the interaction fractions observed throughout the simulation, classified by interaction type: water‐mediated hydrogen bonds (blue), direct hydrogen bonds (green), ionic interactions (pink), and hydrophobic contacts (purple). These visualizations highlight the predominant interactions formed between compound **3p** and key residues within the active sites of the respective enzyme. Detailed numerical data corresponding to these interactions is provided in Table 4. These interactions further support the structural stability and favorable binding conformations of the AChE enzyme complex formed by compounds **3p**.

The temporal stability of these interactions was further validated through a timeline analysis presented in Figure 4E. The total number of contacts between compound **3p** and AChE fluctuated around a mean of 10 distinct interactions, reflecting a continuous and saturated binding profile. The bottom panel of the timeline reveals that the interactions with Trp86, Tyr124, Trp286, Glu292, Tyr337, Phe338, and Tyr341 were maintained with high occupancy throughout the 100 ns simulation. These persistent contacts are indicative of a high‐affinity binding event and suggest that compound **3p** can effectively block the enzymatic activity by remaining tightly anchored within the gorge of the AChE enzyme.

Finally, the physicochemical properties of the ligand during the MD run were monitored, including the radius of gyration (rGyr) and torsion profiles (Figure 4F,G). The rGyr values, which measure the compactness of the ligand, remained relatively stable between 5.5 and 6 Å, suggesting that compound **3p** does not undergo significant unfolding or structural distortion within the binding site. The convergence of rGyr and other parameters such as Molecular Surface Area (MolSA) and Polar Surface Area (PSA) further supports the adoption of a compact and stable conformation. Furthermore, the torsion analysis confirmed that the rotatable bonds of the ligand reached stable equilibrium angles, minimizing internal strain and contributing to the overall thermodynamic favorability of the AChE‐compound **3p** complex.

In conclusion, these quantitative stability and interaction metrics provide unequivocal structural evidence that compound **3p** forms a highly persistent, dual‐site binding complex within AChE, corroborating the nanomolar inhibitory potency observed in vitro. The structural integrity of the complex is rigorously supported by consistent RMSD and RMSF profiles, which remained within acceptable limits throughout the trajectory. Furthermore, the stable rGyr values and the convergence of ligand torsion angles confirm that compound **3p** retains a compact and energetically favorable orientation within the enzymatic pocket. The exceptional stability of the binding is further evidenced by persistent, high‐occupancy interactions with key residues, most notably the strong π‐stacking and cation‐π interactions with Trp86, as well as the robust water‐mediated hydrogen bonding network involving Glu292 and Tyr341. The minimal fluctuation levels observed in the RMSF analysis indicate that the ligand remains tightly anchored within the catalytic gorge, effectively stabilizing the protein framework.

Furthermore, MD data clearly demonstrated that the substituents at the 3rd and 4th positions of compound **3p** are stably anchored within the enzyme's active site through direct hydrogen bonds and water‐mediated hydrogen bonding networks. This finding unequivocally validates the hypothesis generated during the molecular docking studies; specifically, that the presence of polar groups at these positions, capable of hydrogen bonding, is essential for stable binding. These interactions provide a robust structural rationale for the superior in vitro enzyme inhibitory potencies observed for these specific derivatives. Overall, these findings suggest that compound **3p** not only exhibits high initial binding affinity but also retains significant structural stability over time. Consequently, this compound emerges as a highly promising candidate for further optimization and investigation in the development of potent enzyme‐targeted therapeutics for neurodegenerative disorders.

## 3 Materials and Methods

### 3.1 Chemistry

All chemicals were purchased from Sigma‐Aldrich Chemicals Co. (Sigma‐Aldrich Corp., St. Louis, MO, USA). All the reactions were monitored by thin‐layer chromatography (TLC) using silica gel‐60 F_254_ TLC plates (Merck KGaA, Darmstadt, Germany). All melting points (m.p.) were determinated by an Electrothermal IA‐9100 Melting Point Apparatus (Electrothermal Inc., NJ, USA) and were uncorrected. Infrared spectra were recorded on a Shimadzu FT‐IR 8400S ATR spectrometer (Shimadzu Corp., Tokyo, Japan). ^1^H‐NMR and ^13^C‐NMR spectra were recorded in CDCl_3_ using a Bruker Avance 300 MHz spectrometer (Bruker Bioscience, Billerica, MA, USA). Elemental analyses were performed on a LECO CHNS‐932 elemental analyzer (St. Joseph, MI, USA).

#### 3.1.1 Synthesis Procedure of Ethyl 2‐(4‐(2,3,4‐trimethoxybenzyl)piperazin‐1‐yl)acetate [1]

Trimetazidine dihydrochloride was dissolved in acetone (0.5 M), NaHCO_3_ (1.25 equiv.) was added, and the mixture was stirred at room temperature. Ethyl bromoacetate (1.1 equiv.) was then added dropwise. The reaction mixture was stirred overnight at reflux. The reaction mixture was cooled to room temperature. The reaction mixture was filtered and washed with acetone. The filtrate was concentrated. The crude product was purified by silica gel column chromatography to yield pure ethyl ester (compound **1 –** CAS No. 119929‐68‐9) (Ohtaka et al. 1988).

#### 3.1.2 Synthesis Procedure of 2‐[4‐(2,3,4‐trimethoxybenzyl)piperazin‐1‐yl]acetohydrazide [2]

The ethyl ester (compound **1 –** CAS No. 119929‐68‐9) (1.0 equiv.) was dissolved in ethanol. The solution was stirred, and hydrazine monohydrate (3.0−4.0 equiv.) was added dropwise. The reaction mixture was stirred at reflux. The mixture was cooled and concentrated in vacuo. The concentrate was transferred to a separatory funnel containing brine:water made basic (~pH> 12) with NaOH. The aqueous layer was extracted with DCM (x4). The organic layers were dried over sodium sulfate and evaporated on vacuo. The crude product was purified by recrystallization to yield pure hydrazide (compound **2**) (Hsu et al. 2012).

#### 3.1.3 2‐[4‐(2,3,4‐Trimethoxybenzyl)piperazin‐1‐yl]acetohydrazide [2]

White solid, yield: 80%, m.p.: 98°C. TLC (1:1 *v/v*; DCM:Methanol); Rf = 0.25. IR (v_max_, cm^−1^): 3321, 3271, 3215 (N‐H); 3045, 3018 (aromatic C‐H); 2929, 2910, 2874, 2835, 2762 (aliphatic and piperazine C‐H); 1647 (C = O); 1495 (aromatic C = C). ^1^H. NMR (600 MHz, CDCl_3_) *δ* 8.14 (s, 1H, NH), 6.94 (d, *J* = 8.5 Hz, 1H, aromatic protons), 6.62 (d, *J* = 8.5 Hz, 1H, aromatic protons), 3.86 (s, 2H, NH_2_), 3.86–3.83 (m, 9H, O‐CH_3_), 3.46 (s, 2H, Ar‐CH_2_), 3.04 (s, 2H, O = C‐CH_2_), 2.58–2.39 (m, 8H, piperazine protons). ^13^C NMR (75 MHz, decoupled, CDCl_3_) *δ* 170.59 (C = O), 152.95, 152.63, 142.29, 125.08, 123.72, 106.93, 61.22 (O = C‐CH_2_), 60.80 (O‐CH_3_), 60.60 (O‐CH_3_), 56.36 (Ar‐CH_2_ ), 55.96 (O‐CH_3_), 53.79 and 52.88 (piperazine carbons). Elemental analysis: Calcd. for C_16_H_26_N_4_O_4_: C, 56.79; H, 7.74; N, 16.56%; found: C, 56.55; H, 7.73; N, 16.41%.

#### 3.1.4 Synthesis Procedure of Compounds 3a‐t

The hydrazide (compound **2**) was dissolved in hot ethanol (15 mL) and aldehyde derivatives (1.1 equiv.) was added. The reaction mixture was stirred at reflux, monitored by TLC and concentrated in vacuo. All hydrazone derivatives washed with diethyl ether and recrystallized with ethanol to yield hydrazones (compounds **3a‐t**) (Ali et al. 2025).

#### 3.1.5 *N*’‐Benzylidene‐2‐[4‐(2,3,4‐trimethoxybenzyl)piperazin‐1‐yl]acetohydrazide [3a]

Off white solid, yield: 74%, m.p.: 121°C. TLC (1:1 *v/v*; DCM:Methanol); Rf = 0.78. IR (v_max_, cm^−1^): 3425 (N‐H); 3078, 3047 (aromatic C‐H); 2976, 2931, 2829 (aliphatic and piperazine C‐H); 1683 (C = O); 1602 (C = N); 1498 (aromatic C = C). ^1^H NMR (300 MHz, CDCl_3_) *δ* 9.76 (s, 1H, NH), 8.42 (s, 1H, N = CH), 7.55 (dd, *J* = 8.5, 3.5 Hz, 2H, aromatic protons), 7.45–7.28 (m, 3H, aromatic protons), 7.19 (d, *J* = 8.0 Hz, 1H, aromatic protons), 6.70 (d, *J* = 9.0 Hz, 1H, aromatic protons), 3.86–3.84 (m, 9H, O‐CH_3_), 3.42 (s, 2H, Ar‐CH_2_), 3.17 (s, 2H, O = C‐CH_2_), 2.45–2.33 (m, 8H, piperazine protons). ^13^C NMR (150 MHz, CDCl_3_) *δ* 165.11 (C = O), 155.67, 153.04, 152.96, 149.81 (C = N), 141.79, 140.93, 130.03, 129.87, 129.73, 129.55, 129.40, 128.57, 127.76, 127.26, 107.62, 61.30 (O = C‐CH_2_), 60.91 (O‐CH_3_), 58.10 (O‐CH_3_), 57.23 (O‐CH_3_), 56.08 (Ar‐CH_2_ ), 54.18 and 50.32 (piperazine carbons). Elemental Analysis: Calcd. for C_23_H_30_N_4_O_4_: C, 64.77; H, 7.09; N, 13.14%; found: C, 63.51; H, 7.03; N, 12.81%.

#### 3.1.6 *N*’‐(4‐methylbenzylidene)−2‐[4‐(2,3,4‐trimethoxybenzyl)piperazin‐1‐yl]acetohydrazide [3b]

Off white solid, yield: 88%, m.p.: 139°C. TLC (1:1 *v/v*; DCM:Methanol); Rf = 0.80. IR (v_max_, cm^−1^): 3321, 3271 (N‐H); 3045, 3018 (aromatic C‐H); 2929, 2910, 2874 (aliphatic and piperazine C‐H); 1647 (C = O); 1600 (C = N); 1495 (aromatic C = C). ^1^H NMR (300 MHz, CDCl_3_) *δ* 10.06 (s, 1H, NH), 8.13 (s, 1H, N = CH), 7.64 (d, *J* = 8.0 Hz, 2H, aromatic protons), 7.19 (d, *J* = 8.0 Hz, 2H, aromatic protons), 6.98 (d, *J* = 8.5 Hz, 1H, aromatic proton), 6.64 (d, *J* = 8.5 Hz, 1H, aromatic proton), 3.88–3.85 (m, 9H, O‐CH_3_), 3.51 (s, 2H, Ar‐CH_2_), 3.17 (s, 2H, O = C‐CH_2_), 2.61–2.54 (m, 8H, piperazine), 2.36 (s, 3H, Ar‐CH_3_). ^13^C NMR (75 MHz, decoupled, CDCl_3_) *δ* 166.27 (C = O), 153.03, 152.64, 148.53 (C = N), 142.27, 140.94, 130.77, 129.43, 127.72, 125.18, 123.49, 107.01, 61.28 (O = C‐CH_2_), 61.03 (O‐CH_3_), 60.82 (O‐CH_3_), 56.28 (Ar‐CH_2_ ), 55.97 (O‐CH_3_), 53.69 and 52.85 (piperazine carbons), 21.54 (CH_3_). Elemental Analysis: Calcd. for C_24_H_32_N_4_O_4_: C, 65.43; H, 7.32; N, 12.72%; found: C, 65.35; H, 7.21; N, 12.51%.

#### 3.1.7 *N*’‐(4‐fluorobenzylidene)−2‐[4‐(2,3,4‐trimethoxybenzyl)piperazin‐1‐yl]acetohydrazide [3c]

Off white solid, yield: 80%, m.p.: 148°C. TLC (1:1 *v/v*; DCM:Methanol); Rf = 0.79. IR (v_max_, cm^−1^): 3462, 3300 (N‐H); 3165, 3147 (aromatic C‐H); 2972, 2933, 2839 (aliphatic and piperazine C‐H); 1707 (C = O); 1604 (C = N); 1496 (aromatic C = C). ^1^H NMR (300 MHz, CDCl_3_) *δ* 10.12 (s, 1H, NH), 8.20 (s, 1H, N = CH), 7.69 (d, *J* = 8.6 Hz, 2H, aromatic protons), 7.37 (d, *J* = 8.6 Hz, 2H, aromatic protons), 6.98 (d, *J* = 8.5 Hz, 1H, aromatic proton), 6.64 (d, *J* = 8.6 Hz, 1H, aromatic proton), 3.89–3.85 (m, 9H, O‐CH_3_), 3.50 (s, 2H, Ar‐CH_2_), 3.17 (s, 2H, O = C‐CH_2_), 2.60–2.54 (m, 8H, piperazine). ^13^C NMR (75 MHz, decoupled, CDCl_3_) *δ* 166.53 (C = O), 152.81, 147.10 (C = N), 142.29, 136.44, 132.14, 128.93, 125.10, 123.67, 107.00, 106.86, 61.29 (O = C‐CH_2_), 61.06 (O‐CH_3_), 60.83 (O‐CH_3_), 56.37 (O‐CH_3_), 55.98 (Ar‐CH_2_), 53.79 and 52.94 (piperazine carbons). Elemental Analysis: Calcd. for C_23_H_29_FN_4_O_4_: C, 62.15; H, 6.58; N, 12.60%; found: C, 61.78; H, 6.38; N, 12.52%.

#### 3.1.8 *N*’‐(4‐chlorobenzylidene)−2‐[4‐(2,3,4‐trimethoxybenzyl)piperazin‐1‐yl]acetohydrazide [3d]

Off white solid, yield: 82%, m.p.: 140°C. TLC (1:1 *v/v*; DCM:Methanol); Rf = 0.79. IR (v_max_, cm^−1^): 3462, 3300 (N‐H); 3155, 3170 (aromatic C‐H); 2991, 2936, 2825 (aliphatic and piperazine C‐H); 1705 (C = O); 1599 (C = N); 1496 (aromatic C = C). ^1^H NMR (300 MHz, CDCl_3_) *δ* 10.09 (s, 1H, NH), 8.21 (s, 1H, N = CH), 7.74 (dd, *J* = 9.0, 5.5 Hz, 2H, aromatic protons), 7.07 (d, *J* = 9.0 Hz, 2H, aromatic protons), 6.97 (d, *J* = 8.5 Hz, 1H, aromatic proton), 6.64 (d, *J* = 8.5 Hz, 1H, aromatic proton), 3.90–3.86 (m, 9H, O‐CH_3_), 3.52 (s, 2H, Ar‐CH_2_), 3.19 (s, 2H, O = C‐CH_2_), 2.70–2.56 (m, 8H, piperazine). ^13^C NMR (75 MHz, decoupled, CDCl_3_) *δ* 166.47 (C = O), 152.99, 152.62, 147.22 (C = N), 142.29, 129.86, 129.82, 129.64, 129.52, 125.09, 123.68, 116.03, 115.74, 107.00, 61.28 (O = C‐CH_2_), 61.05 (O‐CH_3_), 60.82 (O‐CH_3_), 56.37 (Ar‐CH_2_ ), 55.98 (O‐CH_3_), 53.78 and 52.93 (piperazine carbons). Elemental Analysis: Calcd. for C_23_H_29_ClN_4_O_4_: C, 59.93; H, 6.34; N, 12.15%; found: C, 59.40; H, 6.25; N, 12.05%.

#### 3.1.9 *N*’‐(4‐bromobenzylidene)−2‐[4‐(2,3,4‐trimethoxybenzyl)piperazin‐1‐yl]acetohydrazide [3e]

Off white solid, yield: 85%, m.p.: 138°C. TLC (1:1 *v/v*; DCM:Methanol); Rf = 0.80. IR (v_max_, cm^−1^): 3488, 3300 (N‐H); 3163, 3150 (aromatic C‐H); 2972, 2933, 2827 (aliphatic and piperazine C‐H); 1707 (C = O); 1606 (C = N); 1496 (aromatic C = C). ^1^H NMR (300 MHz, CDCl_3_) *δ* 10.13 (s, 1H, NH), 8.17 (s, 1H, N = CH), 7.62 (d, *J* = 8.6 Hz, 2H, aromatic protons), 7.52 (d, *J* = 8.5 Hz, 2H, aromatic protons), 6.97 (d, *J* = 8.5 Hz, 1H, aromatic proton), 6.64 (d, *J* = 8.5 Hz, 1H, aromatic proton), 3.90–3.84 (m, 9H, 3O‐CH_3_), 3.50 (s, 2H, Ar‐CH_2_), 3.17 (s, 2H, O = C‐CH_2_), 2.63–2.51 (m, 8H, piperazine protons). ^13^C NMR (75 MHz, decoupled, CDCl_3_) *δ* 166.55 (C = O), 153.00, 152.62, 147.17 (C = N), 142.30, 132.58, 131.96, 129.04, 125.10, 124.83, 123.66, 107.01, 61.28 (O = C‐CH_2_), 61.06 (O‐CH_3_), 60.82 (O‐CH_3_), 56.37 (Ar‐CH_2_ ), 55.98 (O‐CH_3_), 53.78 and 52.93 (piperazine carbons). Elemental Analysis: Calcd. for C_23_H_29_BrN_4_O_4_: C, 54.66; H, 5.78; N, 11.09%; found: C, 54.41; H, 5.36; N, 11.01%.

#### 3.1.10 *N*’‐(4‐hydroxybenzylidene)−2‐[4‐(2,3,4‐trimethoxybenzyl)piperazin‐1‐yl]acetohydrazide [3f]

Yellow solid, yield: 79%, m.p.: 126°C. TLC (1:1 *v/v*; DCM:Methanol); Rf = 0.77. IR (v_max_, cm^−1^): 3584 (O‐H); 3470, 3300 (N‐H); 3050, 2987 (aromatic C‐H); 2937, 2821, 2773 (aliphatic and piperazine C‐H); 1647 (C = O); 1602 (C = N); 1494 (aromatic C = C). ^1^H NMR (300 MHz, CDCl_3_) *δ* 10.13 (s, 1H, NH), 7.97 (s, 1H, N = CH), 7.52 (d, *J* = 9.0 Hz, 2H, aromatic protons), 6.96 (d, *J* = 8.5 Hz, 1H, aromatic proton), 6.84 (d, *J* = 8.5 Hz, 2H, aromatic protons), 6.63 (d, *J* = 8.5 Hz, 1H, aromatic proton), 3.88–3.82 (m, 9H, 3O‐CH_3_), 3.50 (s, 2H, Ar‐CH_2_), 3.16 (s, 2H, O = C‐CH_2_), 2.61–2.55 (m, 8H, piperazine protons). ^13^C NMR (75 MHz, decoupled, CDCl_3_) *δ* 166.80 (C = O), 159.79, 153.07, 152.61, 149.25 (C = N), 142.22, 129.63, 125.31, 124.69, 123.29, 107.04, 61.26 (O = C‐CH_2_), 61.12 (O‐CH_3_), 60.82 (O‐CH_3_), 56.33 (Ar‐CH_2_ ), 55.98 (O‐CH_3_), 53.57 and 52.83 (piperazine carbons). Elemental Analysis: Calcd. for C_23_H_30_N_4_O_5_: C, 62.43; H, 6.83; N, 12.66%; found: C, 62.35; H, 6.36; N, 12.45%.

#### 3.1.11 *N*’‐(4‐nitrobenzylidene)−2‐[4‐(2,3,4‐trimethoxybenzyl)piperazin‐1‐yl]acetohydrazide [3g]

Yellow solid, yield: 77%, m.p.: 167°C. TLC (1:1 *v/v*; DCM:Methanol); Rf = 0.80. IR (v_max_, cm^−1^): 3460, 3400 (N‐H); 3101, 3010 (aromatic C‐H); 2949, 2928, 2814 (aliphatic and piperazine C‐H); 1683 (C = O); 1600 (C = N); 1506 (N‐O); 1495 (aromatic C = C). ^1^H NMR (300 MHz, CDCl_3_) *δ* 10.30 (s, 1H, NH), 8.44 (s, 1H, N = CH), 8.25 (d, *J* = 9.0 Hz, 2H, aromatic protons), 7.91 (d, *J* = 9.0 Hz, 2H, aromatic protons), 6.97 (d, *J* = 9.0 Hz, 1H, aromatic proton), 6.64 (d, *J* = 9.0 Hz, 1H, aromatic proton), 3.93–3.81 (m, 9H, 3O‐CH_3_), 3.50 (s, 2H, Ar‐CH_2_), 3.20 (s, 2H, O = C‐CH_2_), 2.62–2.51 (m, 8H, piperazine protons). ^13^C NMR (75 MHz, decoupled, CDCl_3_) *δ* 166.96 (C = O), 153.04, 152.64, 148.69, 145.73 (C = N), 142.33 139.89, 128.15, 125.08, 124.00, 123.63, 107.04, 61.29 (O = C‐CH_2_), 61.12 (O‐CH_3_), 60.82 (O‐CH_3_), 56.37 (Ar‐CH_2_ ), 55.99 (O‐CH_3_), 53.82 and 52.91 (piperazine carbons). Elemental Analysis: Calcd. for C_23_H_29_N_5_O_6_: C, 58.59; H, 6.20; N, 14.85%; found: C, 58.27; H, 6.12; N, 14.78%.

#### 3.1.12 *N*’‐(4‐methoxybenzylidene)−2‐[4‐(2,3,4‐trimethoxybenzyl)piperazin‐1‐yl]acetohydrazide [3h]

Off white solid, yield: 82%, m.p.: 123°C. TLC (1:1 *v/v*; DCM:Methanol); Rf = 0.83. IR (v_max_, cm^−1^): 3446, 3444 (N‐H); 3190, 3080 (aromatic C‐H); 2960, 2937, 2814 (aliphatic and piperazine C‐H); 1689 (C = O); 1604 (C = N); 1496 (aromatic C = C). ^1^H NMR (300 MHz, CDCl_3_) *δ* 10.00 (s, 1H, NH), 8.10 (s, 1H, N = CH), 7.70 (d, *J* = 9.0 Hz, 2H, aromatic protons), 6.98 (d, *J* = 9.0 Hz, 1H, aromatic proton), 6.91 (d, *J* = 9.0 Hz, 2H, aromatic protons), 6.64 (d, *J* = 9.0 Hz, 1H, aromatic proton), 3.90–3.82 (m, 12H, 4O‐CH_3_), 3.50 (s, 2H, Ar‐CH_2_), 3.16 (s, 2H, O = C‐CH_2_), 2.61–2.57 (m, 8H, piperazine protons). ^13^C NMR (75 MHz, decoupled, CDCl_3_) *δ* 166.19 (C = O), 161.57, 152.97, 152.62, 148.20 (C = N), 142.29, 129.34, 126.18, 125.09, 123.74, 114.15, 107.00, 61.29 (O = C‐CH_2_), 61.05 (O‐CH_3_), 60.82 (O‐CH_3_), 56.39 (Ar‐CH_2_ ), 55.98 (O‐CH_3_), 55.37, 53.78 and 52.97 (piperazine carbons). Elemental Analysis: Calcd. for C_24_H_32_N_4_O_5_: C, 63.14; H, 7.07; N, 12.27%; found: C, 62.99; H, 7.00; N, 11.73%.

#### 3.1.13 *N*’‐[4‐(methylthio)benzylidene]−2‐[4‐(2,3,4‐trimethoxybenzyl)piperazin‐1‐yl]acetohydrazide [3i]

Off white solid, yield: 80%, m.p.: 121°C. TLC (1:1 *v/v*; DCM:Methanol); Rf = 0.83. IR (v_max_, cm^−1^): 3446, 3440 (N‐H); 3182, 3080 (aromatic C‐H); 2937, 2920, 2810 (aliphatic and piperazine C‐H); 1687 (C = O); 1597 (C = N); 1492 (aromatic C = C); 1247 (Ar‐C‐S). ^1^H NMR (300 MHz, CDCl_3_) *δ* 10.10 (s, 1H, NH), 8.12 (s, 1H, N = CH), 7.65 (d, *J* = 9.0 Hz, 2H, aromatic protons), 7.22 (d, *J* = 9.0 Hz, 2H, aromatic protons), 6.99 (d, *J* = 9.0 Hz, 1H, aromatic proton), 6.64 (d, *J* = 9.0 Hz, 1H, aromatic proton), 3.88–3.84 (m, 9H, 3O‐CH_3_), 3.54 (s, 2H, Ar‐CH_2_), 3.17 (s, 2H, O = C‐CH_2_), 2.62–2.57 (m, 8H, piperazine protons), 2.49 (s, 3H, S‐CH_3_). ^13^C NMR (75 MHz, decoupled, CDCl_3_) *δ* 166.21 (C = O), 153.16, 152.65, 148.06 (C = N), 142.24, 142.12 130.03, 128.01, 125.73, 125.34, 122.93, 107.04, 61.28 (O = C‐CH_2_), 60.96 (O‐CH_3_), 60.82 (O‐CH_3_), 56.08 (Ar‐CH_2_ ), 55.98 (O‐CH_3_), 53.46 and 52.63 (piperazine carbons), 15.11 (S‐CH_3_). Elemental Analysis: Calcd. for C_24_H_32_N_4_O_4_S: C, 60.99; H, 6.83; N, 11.86 S, 6.78%; found: C, 60.95; H, 6.80; N, 11.49; S, 6.48%.

#### 3.1.14 *N*’‐[4‐(trifluoromethyl)benzylidene]−2‐[4‐(2,3,4‐trimethoxybenzyl)piperazin‐1‐yl]acetohydrazide [3j]

Yellow solid, yield: 75%, m.p.: 119°C. TLC (1:1 *v/v*; DCM:Methanol); Rf = 0.82. IR (v_max_, cm^−1^): 3462, 3444 (N‐H); 3203, 3199 (aromatic C‐H); 2982, 2937, 2810 (aliphatic and piperazine C‐H); 1666 (C = O); 1604 (C = N); 1496 (aromatic C = C); 1049 (C‐F). ^1^H NMR (300 MHz, CDCl_3_) *δ* 10.24 (s, 1H, NH), 8.32 (s, 1H, N = CH), 7.85 (d, *J* = 9.0 Hz, 2H, aromatic protons), 7.64 (d, *J* = 9.0 Hz, 2H, aromatic protons), 6.98 (d, *J* = 9.0 Hz, 1H, aromatic proton), 6.64 (d, *J* = 9.0 Hz, 1H, aromatic proton), 3.89–3.84 (m, 9H, 3O‐CH_3_), 3.54 (s, 2H, Ar‐CH_2_), 3.19 (s, 2H, O = C‐CH_2_), 2.64–2.56 (m, 8H, piperazine protons). ^13^C NMR (75 MHz, decoupled, CDCl_3_) *δ* 166.67 (C = O), 153.13, 152.65, 146.75 (C = N), 142.25, 137.06, 127.79, 125.67, 125.62, 125.29, 123.07, 122.05, 107.02, 61.27 (O = C‐CH_2_), 61.02 (O‐CH_3_), 60.82 (O‐CH_3_), 56.11 (Ar‐CH_2_ ), 55.97 (O‐CH_3_), 53.56 and 52.64 (piperazine carbons). Elemental Analysis: Calcd. for C_24_H_29_F_3_N_4_O_4_: C, 58.29; H, 5.91; N, 11.33%; found: C, 58.26; H, 5.77; N, 11.21%.

#### 3.1.15 *N*’‐[4‐(dimethylamino)benzylidene]−2‐[4‐(2,3,4‐trimethoxybenzyl)piperazin‐1‐yl]acetohydrazide [3k]

Yellow solid, yield: 84%, m.p.: 134°C. TLC (1:1 *v/v*; DCM:Methanol); Rf = 0.83. IR (v_max_, cm^−1^): 3483, 3400 (N‐H); 3190, 3180 (aromatic C‐H); 2999, 2931, 2808 (aliphatic and piperazine C‐H); 1681 (C = O); 1600 (C = N); 1495 (aromatic C = C); 1361 (aromatic amine C‐N). ^1^H NMR (300 MHz, CDCl_3_) *δ* 9.90 (s, 1H, NH), 7.99 (s, 1H, N = CH), 7.63 (d, *J* = 9.0 Hz, 2H, aromatic protons), 6.98 (d, *J* = 9.0 Hz, 1H, aromatic proton), 6.65 (t, *J* = 9.0 Hz 3H, aromatic protons), 3.88–3.84 (m, 9H, 3O‐CH_3_), 3.49 (s, 2H, Ar‐CH_2_), 3.15 (s, 2H, O = C‐CH_2_), 3.01 (s, 6H, N‐(CH_3_)_2_), 2.60–2.53 (m, 8H, piperazine protons). ^13^C NMR (75 MHz, decoupled, CDCl_3_) *δ* 165.80 (C = O), 152.95, 152.62, 151.91, 149.11 (C = N), 142.29, 129.28, 125.10, 123.79, 121.01, 111.61, 107.00, 61.29 (O = C‐CH_2_), 61.04 (O‐CH_3_), 60.82 (O‐CH_3_), 56.40 (Ar‐CH_2_ ), 55.98 (O‐CH_3_), 53.76 and 53.01 (piperazine carbons), 40.16 (N‐(CH_3_)_2_). Elemental Analysis: Calcd. for C_25_H_35_N_5_O_4_: C, 63.94; H, 7.51; N, 14.91%; found: C, 63.72; H, 7.38; N, 14.55%.

#### 3.1.16 *N*’‐(4‐isopropylbenzylidene)−2‐[4‐(2,3,4‐trimethoxybenzyl)piperazin‐1‐yl]acetohydrazide [3l]

Off white solid, yield: 70%, m.p.: 119°C. TLC (1:1 *v/v*; DCM:Methanol); Rf = 0.80. IR (v_max_, cm^−1^): 3468, 3400 (N‐H); 3190, 3138 (aromatic C‐H); 2958, 2933, 2810 (aliphatic and piperazine C‐H); 1689 (C = O); 1600 (C = N); 1495 (aromatic C = C). ^1^H NMR (300 MHz, CDCl_3_) *δ* 10.06 (s, 1H, NH), 8.16 (s, 1H, N = CH), 7.69 (d, *J* = 9.0 Hz, 2H, aromatic protons), 7.26 (d, *J* = 9.0 Hz, 2H, aromatic protons), 6.99 (d, *J* = 9.0 Hz, 1H, aromatic proton), 6.65 (d, *J* = 9.0 Hz, 1H, aromatic proton), 3.90–3.85 (m, 9H, 3O‐CH_3_), 3.52 (s, 2H, Ar‐CH_2_), 3.18 (s, 2H, O = C‐CH_2_), 2.97–2.88 (m, 1H, isopropyl CH), 2.62–2.56 (m, 8H, piperazine protons), 1.26 (d, *J* = 7.0 Hz, 6H, isopropyl HC‐(CH_3_)_2_). ^13^C NMR (150 MHz, APT, CDCl_3_) *δ* 166.27 (C = O), 153.01, 152.65, 151.78, 148.60 (C = N), 142.35, 131.20, 127.82, 126.80, 125.09, 123.71, 107.08, 61.26 (O = C‐CH_2_ ), 61.10 (O‐CH_3_), 60.80 (O‐CH_3_), 56.36 (Ar‐CH_2_ ), 55.99 (O‐CH_3_), 53.76 and 52.94 (piperazine carbons), 34.12 (isopropyl CH), 23.76 (isopropyl CH_3_). Elemental Analysis: Calcd. for C_26_H_36_N_4_O_4_: C, 66.64; H, 7.74; N, 11.96%; found: C, 66.24; H, 7.60; N, 11.59%.

#### 3.1.17 Methyl 4‐[(2‐(2‐(4‐(2,3,4‐trimethoxybenzyl)piperazin‐1‐yl)acetyl)hydrazineylidene)methyl]benzoate [3m]

Off white solid, yield: 85%, m.p.: 135°C. TLC (1:1 *v/v*; DCM:Methanol); Rf = 0.87. IR (v_max_, cm^−1^): 3487, 3400 (N‐H); 3163, 3130 (aromatic C‐H); 2962, 2939, 2810 (aliphatic and piperazine C‐H); 1716, 1687 (C = O); 1600 (C = N); 1495 (aromatic C = C); 1361 (ester C‐O). ^1^H NMR (300 MHz, CDCl_3_) *δ* 10.21 (s, 1H, NH), 8.27 (s, 1H, N = CH), 8.05 (d, *J* = 9.0 Hz, 2H, aromatic protons), 7.81 (d, *J* = 9.0 Hz, 2H, aromatic protons), 6.97 (d, *J* = 9.0 Hz, 1H, aromatic proton), 6.64 (d, *J* = 9.0 Hz, 1H, aromatic proton), 3.92 (s, 3H, ester O‐CH_3_), 3.88–3.84 (m, 9H, 3O‐CH_3_), 3.51 (s, 2H, Ar‐CH_2_), 3.18 (s, 2H, O = C‐CH_2_), 2.71–2.55 (m, 8H, piperazine protons). ^13^C NMR (75 MHz, decoupled, CDCl_3_) *δ* 166.65 (C = O), 166.55 (C = O), 152.03, 152.63, 147.10 (C = N), 142.28, 137.81, 131.57, 129.91, 127.50, 125.15, 123.49, 107.00, 61.28 (O = C‐CH_2_), 61.06 (O‐CH_3_), 60.82 (O‐CH_3_), 56.29 (Ar‐CH_2_ ), 55.97 (O‐CH_3_), 53.72 and 52.83 (piperazine carbons), 52.28 (ester O‐CH_3_). Elemental Analysis: Calcd. for C_25_H_32_N_4_O_6_: C, 61.97; H, 6.66; N, 11.56%; found: C, 61.56; H, 6.36; N, 11.32%.

#### 3.1.18 4‐[(2‐(2‐(4‐(2,3,4‐trimethoxybenzyl)piperazin‐1‐yl)acetyl)hydrazineylidene)methyl]benzoic Acid [3n]

Yellow solid, yield: 69%, m.p.: 115°C. TLC (1:1 *v/v*; DCM:Methanol); Rf = 0.77. IR (v_max_, cm^−1^): 3480 (O‐H); 3460, 3430 (N‐H); 3217, 3196 (aromatic C‐H); 2960, 2933, 2833 (aliphatic and piperazine C‐H); 1681, 1670 (C = O); 1600 (C = N); 1497 (aromatic C = C). ^1^H NMR (300 MHz, CDCl_3_) *δ* 10.36 (s, 1H, NH), 8.08 (s, 1H, N = CH), 8.05 (d, *J* = 9.0 Hz, 2H, aromatic protons), 7.60 (d, *J* = 9.0 Hz, 2H, aromatic protons), 7.20 (d, *J* = 9.0 Hz, 1H, aromatic proton), 6.65 (d, *J* = 9.0 Hz, 1H, aromatic proton), 3.88–3.84 (m, 9H, 3O‐CH_3_), 3.73 (s, 2H, Ar‐CH_2_), 3.27 (s, 2H, O = C‐CH_2_), 2.97–2.79 (m, 8H, piperazine protons). ^13^C NMR (75 MHz, decoupled, CDCl_3_) *δ* 175.75 (C = O), 166.08 (C = O), 154.88, 153.10, 144.37 (C = N), 141.82, 136.27, 130.01, 127.41, 126.85, 119.59, 115.69, 109.11, 61.26 (O = C‐CH_2_), 61.17 (O‐CH_3_), 60.83 (O‐CH_3_), 56.01 (Ar‐CH_2_ ), 55.95 (O‐CH_3_), 52.06 and 51.29 (piperazine carbons). Elemental Analysis: Calcd. for C_24_H_30_N_4_O_6_: C, 61.26; H, 6.43; N, 11.91%; found: C, 61.04; H, 6.23; N, 11.64%.

#### 3.1.19 *N*’‐(2,4‐dihydroxybenzylidene)−2‐[4‐(2,3,4‐trimethoxybenzyl)piperazin‐1‐yl]acetohydrazide [3o]

Orange solid, yield: 66%, m.p.: 116°C. TLC (1:1 *v/v*; DCM:Methanol); Rf = 0.78. IR (v_max_, cm^−1^): 3460 (O‐H); 3300, 3217 (N‐H); 3188, 3132 (aromatic C‐H); 2991, 2937, 2833 (aliphatic and piperazine C‐H); 1680 (C = O); 1604 (C = N); 1495 (aromatic C = C); 1219 (Ar‐C‐O). ^1^H NMR (300 MHz, CDCl_3_) *δ* 9.59 (s, 1H, NH), 8.12 (s, 1H, N = CH), 6.96 (d, *J* = 8.5 Hz, 2H, aromatic protons), 6.62 (d, *J* = 9.0 Hz, 1H, aromatic proton), 6.40–6.34 (m, 2H, aromatic protons), 3.86–3.81 (m, 9H, 3O‐CH_3_), 3.67 (s, 2H, Ar‐CH_2_), 3.15 (s, 2H, O = C‐CH_2_), 2.76–2.64 (m, 8H, piperazine protons). ^13^C NMR (75 MHz, decoupled, CDCl_3_) *δ* 177.64 (C = O), 163.44 and 161.23 (Ar‐C‐OH), 160.27, 153.80, 152.69, 142.00 (C = N), 136.40, 136.27, 126.10, 124.99, 120.26, 109.98, 107.18, 103.58, 61.24 (O = C‐CH_2_), 60.95 (O‐CH_3_), 60.83 (O‐CH_3_), 55.97 (Ar‐CH_2_ ), 55.49 (O‐CH_3_), 52.41 and 51.88 (piperazine carbons). Elemental Analysis: Calcd. for C_23_H_30_N_4_O_6_: C, 60.25; H, 6.60; N, 12.22%; found: C, 60.22; H, 6.36; N, 12.15%.

#### 3.1.20 *N*’‐(3,4‐dihydroxybenzylidene)−2‐[4‐(2,3,4‐trimethoxybenzyl)piperazin‐1‐yl]acetohydrazide [3p]

Yellow solid, yield: 67%, m.p.: 108°C. TLC (1:1 *v/v*; DCM:Methanol); Rf = 0.78. IR (v_max_, cm^−1^): 3460 (O‐H); 3446, 3300 (N‐H); 3225, 3194 (aromatic C‐H); 2999, 2937, 2827 (aliphatic and piperazine C‐H); 1681 (C = O); 1600 (C = N); 1497 (aromatic C = C); 1201 (Ar‐C‐O). ^1^H NMR (300 MHz, CDCl_3_) *δ* 9.59 (s, 1H, NH), 7.84 (s, 1H, N = CH), 6.94 (dd, *J* = 8.5, 4.5 Hz, 1H, aromatic proton), 6.74 (d, *J* = 8.5 Hz, 3H, aromatic protons), 6.55 (d, *J* = 8.5, 1H, aromatic proton), 3.90–3.75 (m, 9H, 3O‐CH_3_), 3.67 (s, 2H, Ar‐CH_2_), 3.13 (s, 2H, O = C‐CH_2_), 2.90–2.54 (m, 8H, piperazine protons). ^13^C NMR (75 MHz, decoupled, CDCl_3_) *δ* 166.71 (C = O), 163.48 and 160.96 (Ar‐C‐OH), 159.98, 153.96, 152.67, 148.68, 145.53 (C = N), 141.89, 125.27, 119.98, 115.69, 107.18, 104.19, 61.19 (O = C‐CH_2_), 61.04 (O‐CH_3_), 60.79 (O‐CH_3_), 55.95 (Ar‐CH_2_ ), 55.66 (O‐CH_3_), 51.92 and 51.80 (piperazine carbons). Elemental Analysis: Calcd. for C_23_H_30_N_4_O_6_: C, 60.25; H, 6.60; N, 12.22%; found: C, 59.86; H, 6.23; N, 11.77%.

#### 3.1.21 *N*’‐(4‐methoxy‐3‐nitrobenzylidene)−2‐[4‐(2,3,4‐trimethoxybenzyl)piperazin‐1‐yl]acetohydrazide [3q]

Off white solid, yield: 77%, m.p.: 178°C. TLC (1:1 *v/v*; DCM:Methanol); Rf = 0.77. IR (v_max_, cm^−1^): 3400, 3350 (N‐H); 3184, 3155 (aromatic C‐H); 3014, 2924, 2820 (aliphatic and piperazine C‐H); 1688 (C = O); 1604 (C = N); 1533 (N‐O); 1497 (aromatic C = C); 1273 (Ar‐C‐O). ^1^H NMR (300 MHz, CDCl_3_) *δ* 10.17 (s, 1H, NH), 8.25 (s, 1H, N = CH), 8.12 (d, *J* = 2.0 Hz, 1H, aromatic proton), 8.03 (dd, *J* = 9.0, 2.0 Hz, 1H, aromatic proton), 7.12 (d, *J* = 9.0 Hz, 1H, aromatic proton), 6.97 (d, *J* = 9.0 Hz, 1H, aromatic proton), 6.64 (d, *J* = 9.0 Hz, 1H, aromatic proton), 3.89 (s, 3H, O‐CH_3_, from aldehyde), 3.88–3.84 (m, 9H, 3O‐CH_3_), 3.50 (s, 2H, Ar‐CH_2_), 3.17 (s, 2H, O = C‐CH_2_), 2.64–2.51 (m, 8H, piperazine protons). ^13^C NMR (75 MHz, decoupled, CDCl_3_) *δ* 166.64 (C = O), 154.26, 153.01, 152.63, 145.65 (C = N), 142.29, 139.63, 132.53, 126.53, 125.11, 125.07, 123.64, 113.83, 107.00, 61.28 (O = C‐CH_2_), 61.05 (O‐CH_3_), 60.82 (O‐CH_3_), 56.78 (Ar‐CH_2_ ), 56.37, 55.98 (O‐CH_3_), 53.77 and 52.91 (piperazine carbons). Elemental Analysis: Calcd. for C_24_H_31_N_5_O_7_: C, 57.48; H, 6.23; N, 13.96%; found: C, 57.27; H, 6.18; N, 13.69%.

#### 3.1.22 *N*’‐(4‐hydroxy‐3‐methoxybenzylidene)−2‐[4‐(2,3,4‐trimethoxybenzyl)piperazin‐1‐yl]acetohydrazide [3r]

Off white solid, yield: 83%, m.p.: 162°C. TLC (1:1 *v/v*; DCM:Methanol); Rf = 0.70. IR (v_max_, cm^−1^): 3470 (O‐H); 3400, 3390 (N‐H); 3196, 3160 (aromatic C‐H); 3034, 2972, 2827 (aliphatic and piperazine C‐H); 1672 (C = O); 1597 (C = N); 1497 (aromatic C = C); 1273 (Ar‐C‐O). ^1^H NMR (300 MHz, CDCl_3_) *δ* 10.13 (s, 1H, NH), 8.05 (s, 1H, N = CH), 7.48 (d, *J* = 2.0 Hz, 1H, aromatic proton), 7.06–6.94 (m, 2H, aromatic protons), 6.89 (d, *J* = 8.0 Hz, 1H, aromatic proton), 6.64 (d, *J* = 9.0 Hz, 1H, aromatic proton), 5.88 (s, 1H, OH), 3.91 (s, 3H, O‐CH_3_, from aldehyde), 3.89–3.81 (m, 9H, 3O‐CH_3_), 3.56 (s, 2H, Ar‐CH_2_), 3.17 (s, 2H, O = C‐CH_2_), 2.65–2.59 (m, 8H, piperazine protons). ^13^C NMR (75 MHz, decoupled, CDCl_3_) *δ* 166.12 (C = O), 153.22, 152.67, 148.90, 148.57, 147.29 (C = N), 142.20, 125.85, 125.47, 123.70, 122.59, 114.26, 107.91, 107.05, 61.26 (O = C‐CH_2_), 61.13 (O‐CH_3_), 60.82 (O‐CH_3_), 56.16 (Ar‐CH_2_), 55.98 (O‐CH_3_), 55.91, 53.31 and 52.46 (piperazine carbons). Elemental Analysis: Calcd. for C_24_H_32_N_4_O_6_: C, 61.00; H, 6.83; N, 11.86%; found: C, 60.89; H, 6.73; N, 11.58%.

#### 3.1.23 *N*’‐[(5‐nitrothiophen‐2‐yl)methylene]−2‐[4‐(2,3,4‐trimethoxybenzyl)piperazin‐1‐yl]acetohydrazide [3s]

Green solid, yield: 81%, m.p.: 175°C. TLC (1:1 *v/v*; DCM:Methanol); Rf = 0.71. IR (v_max_, cm^−1^): 3462, 3300 (N‐H); 3252, 3105 (aromatic C‐H); 3010, 2933, 2818 (aliphatic and piperazine C‐H); 1683 (C = O); 1600 (C = N); 1495 (aromatic C = C); 1429 (N‐O). ^1^H NMR (300 MHz, CDCl_3_) *δ* 10.25 (s, 1H, NH), 9.08 (s, 1H, N = CH), 7.84 (d, *J* = 4.5 Hz, 1H, thiophene aromatic proton), 7.19 (d, *J* = 4.5 Hz, 1H, thiophene aromatic proton), 6.97 (d, *J* = 9.0 Hz, 1H, aromatic proton), 6.64 (d, *J* = 9.0 Hz, 1H, aromatic proton), 3.91–3.81 (m, 9H, 3O‐CH_3_), 3.50 (s, 2H, Ar‐CH_2_), 3.16 (s, 2H, O = C‐CH_2_), 2.63–2.51 (m, 8H, piperazine protons). ^13^C NMR (150 MHz, APT, CDCl_3_) *δ* 167.58 (C = O), 153.05 (thiophene C‐NO_2_), 152.65, 152.52, 146.38 (C = N), 142.94, 142.34, 128.59, 127.90, 125.12, 123.58 (Ar‐C‐CH_2_), 107.03, 61.33 (O = C‐CH_2_ ), 61.26 (O‐CH_3_), 60.81 (O‐CH_3_), 56.36 (Ar‐CH_2_ ), 55.99 (O‐CH_3_), 53.79 and 52.82 (piperazine carbons). Elemental Analysis: Calcd. for C_21_H_27_N_5_O_6_S: C, 52.82; H, 5.70; N, 14.67; S, 6.71%; found: C, 52.66; H, 5.65; N, 14.48; S, 6.64%.

#### 3.1.24 *N*’‐(pyridin‐4‐ylmethylene)−2‐[4‐(2,3,4‐trimethoxybenzyl)piperazin‐1‐yl]acetohydrazide [3t]

Yellow solid, yield: 79%, m.p.: 141°C. TLC (1:1 *v/v*; DCM:Methanol); Rf = 0.68. IR (v_max_, cm^−1^): 3400, 3390 (N‐H); 3196, 3178 (aromatic C‐H); 3057, 2997, 2804 (aliphatic and piperazine C‐H); 1693 (C = O); 1597 (C = N); 1493 (aromatic C = C). ^1^H NMR (300 MHz, CDCl_3_) *δ* 10.29 (s, 1H, NH), 8.65 (d, *J* = 6.0 Hz, 2H, pyridine aromatic protons), 8.30 (s, 1H, N = CH), 7.59 (d, *J* = 6.0 Hz, 2H, pyridine aromatic protons), 6.97 (d, *J* = 9.0 Hz, 1H, aromatic protons), 6.63 (d, *J* = 9.0 Hz, 1H, aromatic protons), 3.92–3.80 (m, 9H, 3O‐CH_3_), 3.50 (s, 2H, Ar‐CH_2_), 3.18 (s, 2H, O = C‐CH_2_), 2.65–2.51 (m, 8H, piperazine protons). ^13^C NMR (150 MHz, APT, CDCl_3_) *δ* 166.93 (C = O), 153.05, 152.63, 150.35, 145.90 (C = N), 142.32, 141.13, 125.1, 123.54, 121.31, 107.05, 61.27 (O = C‐CH_2_), 61.11 (O‐CH_3_), 60.81 (O‐CH_3_), 56.32 (Ar‐CH_2_ ), 55.99 (O‐CH_3_), 53.76 and 52.84 (piperazine carbons). Elemental Analysis: Calcd. for C_22_H_29_N_5_O_4_: C, 61.81; H, 6.84; N, 16.38%; found: C, 61.68; H, 6.62; N, 16.32%.

### 3.2 In Vitro ChE Enzymes Inhibition Assay

Based on the adapted Ellman method described in our previous research, the inhibitory effects of the synthesized compounds on AChE and BChE enzymes were assessed (Ellman et al. 1961; Sağlık et al. 2022; Tok et al. 2019). The enzymes employed in this study were human AChE (CAS No. 9000‐81‐1) and human BChE (CAS No. 9001‐08‐5).

### 3.3 Cytotoxicity Test

The in vitro cytotoxic profiles of the synthesized derivatives were evaluated using the NIH/3T3 mouse embryonic fibroblast cell line (ATCC CRL‐1658). Cells were cultured in 96‐well microplates at an initial seeding density of 1 × 10^4^ cells per well and maintained under standard physiological incubation conditions following established ATCC protocols. Cellular viability was quantitatively determined via the standard MTT colorimetric assay in accordance with previously reported procedures (Sağlık et al. 2022; Tok et al. 2019).

### 3.4 In Silico Studies

#### 3.4.1 Molecular Docking Studies

A structure‐based in silico docking approach was employed to identify potential binding sites and interaction points of the synthesized compounds, which were found to be effective against the active site of AChE enzyme. To achieve this, protein‐ligand interaction analysis was carried out using the crystal structure of AChE (PDB: 4EY7) (Cheung et al. 2012). The docking protocol followed was consistent with the methodology described in our previous studies (Sağlık et al. 2022; Tok et al. 2019).

#### 3.4.2 MDS Studies

MDS were conducted using the *Maestro Desmond* interface program (Schrödinger Release 2020‐3, Desmond, 2020‐3, Desmond. 2020.). A 100 ns simulation period was employed to assess the stability of the compounds identified as promising in the in vitro assays, in conjunction with the docking results. The system setup, execution of the MDS, and subsequent interaction analysis were performed following the methodology outlined in our prior works (Sağlık et al. 2022; Tok et al. 2019).

## 4 Conclusion

Currently, donepezil plays an important role in the treatment of AD as a selective and effective AChE inhibitor. Based on the structure of donepezil, there is a continuing need for new molecules that serve as alternatives to it. For this purpose, a new set of trimetazidine‐derived hydrazone analogues was synthesized and evaluated for cholinesterase inhibition, revealing a clear preference for AChE over BChE across the series. Most compounds displayed pronounced AChE selectivity, whereas BChE inhibition was weak or negligible. Among them, compounds **3p** and **3r** emerged as the leading candidates, exhibiting IC_50_ values closest to donepezil (approximately **1.4‐fold** and **1.6‐fold** less potent, respectively) and thereby representing optimized scaffolds with near donepezil‐like AChE inhibition under the applied assay conditions. Additional potent members (**3q, 3f, 3m, 3h,** and **3n**) also retained sub‐0.15 µM AChE inhibitory activity while maintaining selectivity. Computational studies further supported these findings: docking suggested that **3p** and **3r** adopt donepezil‐like binding modes spanning both the CAS and the PAS, reinforced by π–π contacts with Trp286 and Trp86, as well as π–cation interactions involving the protonated piperazine nitrogen and aromatic residues (Trp86, Tyr337, Tyr341). Hydrogen‐bonding contributions, including interactions involving the carbonyl/NH‐containing linker region (notably with Phe295 and Ser293), were also consistent with stabilized enzyme–ligand complexes. MDS of the AChE–**3p** complex corroborated the persistence of a stable interaction network, aligning with the in vitro activity trend. Overall, these results identify **3p** (and closely **3r**) as primary lead structures for further optimization and validation, and they underscore the potential of this hydrazone framework to deliver highly selective AChE inhibitors. As a critical next step for biological validation, our forthcoming studies will focus on comprehensively evaluating these selected lead candidates in in vitro cellular AD models, including cytotoxicity and neuroprotection assays. Establishing their cellular safety and efficacy profiles will be essential prior to advancing to *in vivo* translational studies.

## Author Contributions

All authors contributed to the study conception and design. Methodology, data curation, conceptualization and analysis were perfomed by [Muhammed Oğuzhan Doğan], [Begüm Nurpelin Sağlık Özkan], [Abdullah Burak Karaduman], [Sevgi Karakuş], [Yusuf Özkay], [Zafer Asım Kaplancıklı] and [Fatih Tok]. The original draft of the manuscript was written by [Muhammed Oğuzhan Doğan], [Begüm Nurpelin Sağlık Özkan], [Abdullah Burak Karaduman], [Sevgi Karakuş], [Yusuf Özkay], [Zafer Asım Kaplancıklı] and [Fatih Tok]. All authors read and approved the final manuscript.

## Conflicts of Interest

The authors declare no conflicts of interest.

## Supporting information


Supporting File

## Data Availability

Data will be made available on request.

## References

[ddr70382-bib-0001] Acar Cevik, U. , B. N. Saglik , S. Levent , et al. 2019. “Synthesis and AChE‐Inhibitory Activity of New Benzimidazole Derivatives.” Molecules 24, no. 5: 861. 10.3390/molecules24050861. 30823470 PMC6429502

[ddr70382-bib-0002] Akhtar, J. , A. A. Khan , Z. Ali , R. Haider , and M. Shahar Yar . 2017. “Structure‐Activity Relationship (SAR) Study and Design Strategies of Nitrogen‐Containing Heterocyclic Moieties for Their Anticancer Activities.” European Journal of Medicinal Chemistry 125: 143–189. 10.1016/j.ejmech.2016.09.023. 27662031

[ddr70382-bib-0003] Ali, S. H. , D. Osmaniye , and Z. A. Kaplancıklı . 2025. “Synthesis and Biological Evaluation of Novel Hydrazone Derivatives for the Treatment of Alzheimer's Disease.” RSC Advances 15, no. 53: 45729–45743. 10.1039/D5RA05755H. 41280213 PMC12637020

[ddr70382-bib-0004] Almaghrabi, M. 2025. “Multitarget‐Directed Ligands for Alzheimer's Disease: Recent Novel MTDLs and Mechanistic Insights.” Pharmaceuticals 18, no. 11: 1685. 10.3390/ph18111685. 41304930 PMC12655775

[ddr70382-bib-0005] Ateş, İ. O. , A. E. Evren , B. N. Sağlik , and L. Yurttaş . 2021. “New Indane Derivatives Containing 2‐Hydrazinothiazole as Potential Acetylcholinesterase and Monoamine Oxidase‐B Inhibitors.” Zeitschrift Für Naturforschung C 76, no. 9–10: 417–424. 10.1515/znc-2021-0058. 34047146

[ddr70382-bib-0006] Bajda, M. , A. Więckowska , M. Hebda , N. Guzior , C. Sotriffer , and B. Malawska . 2013. “Structure‐Based Search for New Inhibitors of Cholinesterases.” International Journal of Molecular Sciences 14, no. 3: 5608–5632. 10.3390/ijms14035608. 23478436 PMC3634507

[ddr70382-bib-0007] Barbuceanu, S. F. , and O. T. Olaru . 2025. “Synthesis and Evaluation of Biologically Active Compounds From Heterocycles Class.” Molecules 30, no. 2: 394. 10.3390/molecules30020394. 39860263 PMC11768012

[ddr70382-bib-0008] Behl, T. , D. Kaur , A. Sehgal , et al. 2021. “Role of Monoamine Oxidase Activity in Alzheimer's Disease: An Insight Into the Therapeutic Potential of Inhibitors.” Molecules 26, no. 12: 3724. 10.3390/molecules26123724. 34207264 PMC8234097

[ddr70382-bib-0010] Celebioglu, H. U. , Y. Erden , F. Hamurcu , et al. 2021. “Cytotoxic Effects, Carbonic Anhydrase Isoenzymes, α‐Glycosidase and Acetylcholinesterase Inhibitory Properties, and Molecular Docking Studies of Heteroatom‐Containing Sulfonyl Hydrazone Derivatives.” Journal of Biomolecular Structure & Dynamics 39, no. 15: 5539–5550. 10.1080/07391102.2020.1792345. 32691677

[ddr70382-bib-0011] Cheung, J. , M. J. Rudolph , F. Burshteyn , et al. 2012. “Structures of Human Acetylcholinesterase in Complex With Pharmacologically Important Ligands.” Journal of Medicinal Chemistry 55, no. 22: 10282–10286. 10.1021/jm300871x. 23035744

[ddr70382-bib-0012] Colovic, M. B. , D. Z. Krstic , T. D. Lazarevic‐Pasti , A. M. Bondzic , and V. M. Vasic . 2013. “Acetylcholinesterase Inhibitors: Pharmacology and Toxicology.” Current neuropharmacology 11, no. 3: 315–335. 10.2174/1570159X11311030006. 24179466 PMC3648782

[ddr70382-bib-0013] Costanzo, P. , L. Cariati , D. Desiderio , et al. 2016. “Design, Synthesis, and Evaluation of Donepezil‐Like Compounds as AChE and BACE‐1 Inhibitors.” ACS Medicinal Chemistry Letters 7, no. 5: 470–475. 10.1021/acsmedchemlett.5b00483. 27190595 PMC4867475

[ddr70382-bib-0014] DeTure, M. A. , and D. W. Dickson . 2019. “The Neuropathological Diagnosis of Alzheimer's Disease.” Molecular Neurodegeneration 14, no. 1: 32. 10.1186/s13024-019-0333-5. 31375134 PMC6679484

[ddr70382-bib-0015] Ellman, G. L. , K. D. Courtney , V. Andres, Jr. , and R. M. Featherstone . 1961. “A New and Rapid Colorimetric Determination of Acetylcholinesterase Activity.” Biochemical Pharmacology 7, no. 2: 88–95. 10.1016/0006-2952(61)90145-9. 13726518

[ddr70382-bib-0016] van Greunen, D. G. , W. Cordier , M. Nell , et al. 2017. “Targeting Alzheimer's Disease by Investigating Previously Unexplored Chemical Space Surrounding the Cholinesterase Inhibitor Donepezil.” European Journal of Medicinal Chemistry 127: 671–690. 10.1016/j.ejmech.2016.10.036. 27823887

[ddr70382-bib-0017] Hsu, D. C. , H. S. Roth , D. C. West , et al. 2012. “Parallel Synthesis and Biological Evaluation of 837 Analogues of Procaspase‐Activating Compound 1 (PAC‐1).” ACS Combinatorial Science 14, no. 1: 44–50. 10.1021/co2001372. 22007686 PMC3253983

[ddr70382-bib-0018] Hussein, W. , B. N. Sağlık , S. Levent , et al. 2018. “Synthesis and Biological Evaluation of New Cholinesterase Inhibitors for Alzheimer's Disease.” Molecules 23, no. 8: 2033. 10.3390/molecules23082033. 30110946 PMC6222329

[ddr70382-bib-0019] Ibrahim, M. , M. Ali , S. A. Halim , et al. 2025. “Synthesis, In‐Vitro Evaluation and In Silico Analysis of New Anticholinesterase Inhibitors Based on Sulfinylbis (Acylhydrazones) Scaffolds.” Journal of Molecular Structure 1334: 141796. 10.1016/j.molstruc.2025.141796.

[ddr70382-bib-0020] Jamal, R. , M. A. Shaikh , M. Taleuzzaman , et al. 2025. “Key Biomarkers in Alzheimer's Disease: Insights for Diagnosis and Treatment Strategies.” Journal of Alzheimer's Disease 105, no. 3: 679–696. 10.1177/13872877251330500. 40255041

[ddr70382-bib-0021] Jampilek, J. 2019. “Heterocycles in Medicinal Chemistry.” Molecules 24, no. 21: 3839. 10.3390/molecules24213839. 31731387 PMC6864827

[ddr70382-bib-0022] Kekeçmuhammed, H. , M. Tapera , E. Aydoğdu , et al. 2023. “Synthesis, Biological Activity Evaluation and Molecular Docking of Imidazole Derivatives Possessing Hydrazone Moiety.” Chemistry & Biodiversity 20, no. 6: e202200886. 10.1002/cbdv.202200886. 37132191

[ddr70382-bib-0023] Kucukoglu, K. , H. I. Gul , P. Taslimi , I. Gulcin , and C. T. Supuran . 2019. “Investigation of Inhibitory Properties of Some Hydrazone Compounds on hCA I, hCA II and AChE Enzymes.” Bioorganic Chemistry 86: 316–321. 10.1016/j.bioorg.2019.02.008. 30743172

[ddr70382-bib-0024] Li, Q. , H. Yang , Y. Chen , and H. Sun . 2017. “Recent Progress in the Identification of Selective Butyrylcholinesterase Inhibitors for Alzheimer's Disease.” European Journal of Medicinal Chemistry 132: 294–309. 10.1016/j.ejmech.2017.03.062. 28371641

[ddr70382-bib-0025] Luque, F. J. , and D. Muñoz‐Torrero . 2024. “Acetylcholinesterase: A Versatile Template to Coin Potent Modulators of Multiple Therapeutic Targets.” Accounts of Chemical Research 57, no. 4: 450–467. 10.1021/acs.accounts.3c00617. 38333993 PMC10882973

[ddr70382-bib-0026] Makhaeva, G. F. , S. V. Lushchekina , N. P. Boltneva , et al. 2015. “Conjugates of γ‐Carbolines and Phenothiazine as New Selective Inhibitors of Butyrylcholinesterase and Blockers of NMDA Receptors for Alzheimer Disease.” Scientific Reports 5, no. 1: 13164. 10.1038/srep13164. 26281952 PMC4642525

[ddr70382-bib-0027] Medeiros, J. 2025. “Phenolic Compounds as Anti‐Alzheimer's Disease Agents.” Exploration of Neuroscience 4: 100693. 10.37349/en.2025.100693.

[ddr70382-bib-0028] Ohtaka, H. , K. Yoshida , K. Suzuki , K. Shimohara , S. Tajima , and K. Ito . 1988. “Benzylpiperazine Derivatives. X. Syntheses and Structure—Antiulcer Activity Relationship of 1‐benzyl‐4‐piperazineacetic Acid Esters.” Chemical & Pharmaceutical Bulletin 36, no. 12: 4825–4833. 10.1248/cpb.36.4825. 3246043

[ddr70382-bib-0029] Pandolfi, F. , D. De Vita , M. Bortolami , et al. 2017. “New Pyridine Derivatives as Inhibitors of Acetylcholinesterase and Amyloid Aggregation.” European Journal of Medicinal Chemistry 141: 197–210. 10.1016/j.ejmech.2017.09.022. 29031067

[ddr70382-bib-0031] Raphael, D. L. 2025. “Neuropsychiatric Symptoms in Mild Cognitive Impairment and Early Alzheimer's Disease: Clinical Pattern and Diagnostic Implications.” AIMS Neuroscience 12, no. 4: 676–705. 10.3934/Neuroscience.2025032. 41523330 PMC12782946

[ddr70382-bib-0032] Rathod, N. , K. R. Thombre , K. R. Gupta , and M. J. Umekar . 2026. “Piperazine as a Privileged Scaffold in Medicinal Chemistry: Biological Activities, Synthetic Strategies, and Multitarget Drug Design.” Chemical Papers 80: 8481–8512. 10.1007/s11696-026-04818-1.

[ddr70382-bib-0033] Saxena, A. , A. M. G. Redman , X. Jiang , O. Lockridge , and B. P. Doctor . 1997. “Differences in Active Site Gorge Dimensions of Cholinesterases Revealed by Binding of Inhibitors to Human Butyrylcholinesterase.” Biochemistry 36, no. 48: 14642–14651. 10.1021/bi971425. 9398183

[ddr70382-bib-0034] Sağlık, B. N. , S. Levent , D. Osmaniye , et al. 2022. “Design, Synthesis, and In Vitro and In Silico Approaches of Novel Indanone Derivatives as Multifunctional Anti‐Alzheimer Agents.” ACS Omega 7, no. 50: 47378–47404. 10.1021/acsomega.2c06906. 36570177 PMC9774391

[ddr70382-bib-0035] Schrödinger Release 2020‐3, Desmond . 2020.

[ddr70382-bib-0036] Sharma, S. , M. Gupta , and S. Sharma . 2025. “Exploring Thiophene‐Based Pharmacophores as Emerging Therapeutics for Neurodegenerative Disorders.” Critical Reviews in Analytical Chemistry. 1–29. 10.1080/10408347.2025.2554239. 40916690

[ddr70382-bib-0037] Sirinzade, H. , N. Faydali , M. T. Muhammed , and E. Dilek . 2026. “Synthesis of New Halo‐Substituted Quinolinecarboxaldehyde‐Hydrazone Derivatives, Structural Illuminations, Investigation of Effects on Acetylcholinesterase Activity and Molecular Modeling Studies.” Journal of Molecular Structure 1351: 144337. 10.1016/j.molstruc.2025.144337.

[ddr70382-bib-0038] Sugimoto, H. , Y. Yamanish , Y. Iimura , and Y. Kawakami . 2000. “Donepezil Hydrochloride (E2020) and Other Acetylcholinesterase Inhibitors.” Current Medicinal Chemistry 7, no. 3: 303–339. 10.2174/0929867003375191. 10637367

[ddr70382-bib-0039] Thal, D. R. , K. Poesen , R. Vandenberghe , and S. De Meyer . 2025. “Alzheimer's Disease Neuropathology and its Estimation with Fluid and Imaging Biomarkers.” Molecular Neurodegeneration 20: 33. 10.1186/s13024-025-00819-y. 40087672 PMC11907863

[ddr70382-bib-0040] Tok, F. , B. Koçyiğit‐Kaymakçıoğlu , B. N. Sağlık , S. Levent , Y. Özkay , and Z. A. Kaplancıklı . 2019. “Synthesis and Biological Evaluation of New Pyrazolone Schiff Bases as Monoamine Oxidase and Cholinesterase Inhibitors.” Bioorganic Chemistry 84: 41–50. 10.1016/j.bioorg.2018.11.016. 30481645

[ddr70382-bib-0041] Yamali, C. , F. S. Engin , S. Bilginer , et al. 2021. “Phenothiazine‐Based Chalcones as Potential Dual‐Target Inhibitors Toward Cholinesterases (AChE, BuChE) and Monoamine Oxidases (MAO‐A, MAO‐B).” Journal of Heterocyclic Chemistry 58, no. 1: 161–171. 10.1002/jhet.4156.

[ddr70382-bib-0042] Zahedi, N. A. , M. Mohammadi‐Khanaposhtani , P. Rezaei , et al. 2023. “Dual Functional Cholinesterase and Carbonic Anhydrase Inhibitors for the Treatment of Alzheimer's Disease: Design, Synthesis, In Vitro, and in Silico Evaluations of Coumarin‐Dihydropyridine Derivatives.” Journal of Molecular Structure 1276: 134767. 10.1016/j.molstruc.2022.134767.

