# Supplementary material for: Design, Synthesis and Molecular Docking Studies of Some Novel Hydrazone Derivatives Based on Trimetazidine as Selective and Potent AChE Inhibitors
Source: Drug Dev Res. 2026 Sep 14;87(7):e70382. doi: 10.1002/ddr.70382 (PMC13572953; doi:10.1002/ddr.70382)
Supplement: Supplementary file 1 [file DDR-87-e70382-s001.docx]

**Design, synthesis and molecular docking studies of some novel hydrazone derivatives based on trimetazidine as selective and potent AChE inhibitors**

**Muhammed Oğuzhan Doğan^a,b^, Begüm Nurpelin Sağlık Özkan^c,d,^*, Abdullah Burak Karaduman^e^, Sevgi Karakuş^f^, Yusuf Özkay^c,d^, Zafer Asım Kaplancıklı^c,d^, Fatih Tok^a,^***

^a^ Department of Pharmaceutical Chemistry, Faculty of Pharmacy, Marmara University, 34854, İstanbul, Türkiye

^b^ Department of Pharmaceutical Chemistry, Institute of Health Sciences, Marmara University, İstanbul, Türkiye

^c^ Department of Pharmaceutical Chemistry, Faculty of Pharmacy, Anadolu University, 26470, Eskişehir, Türkiye

^d^ Central Research Laboratory (MERLAB), Faculty of Pharmacy, Anadolu University, 26470, Eskişehir, Türkiye

^e^ Department of Pharmaceutical Toxicology, Faculty of Pharmacy, Anadolu University, Eskişehir, Türkiye

^f^ Department of Pharmaceutical Chemistry, Faculty of Pharmacy, Istanbul Aydin University, 34295, İstanbul, Türkiye

* Corresponding authors: [bnsaglik@anadolu.edu.tr](mailto:bnsaglik@anadolu.edu.tr); fatih.tok@marmara.edu.tr

**LIST OF CONTENTS**

**Figure S1.** IR spectrum for **2**
**Figure S2.** ^1^H NMR spectrum for **2**
**Figure S3.** ^13^C NMR spectrum for **2**
**Figure S4.** IR spectrum for **3a**
**Figure S5.** ^1^H NMR spectrum for **3a**
**Figure S6.** ^13^C NMR spectrum for **3a**
**Figure S7.** IR spectrum for **3b**
**Figure S8.** ^1^H NMR spectrum for **3b**
**Figure S9.** ^13^C NMR spectrum for **3b**
**Figure S10.** IR spectrum for **3c**
**Figure S11.** ^1^H NMR spectrum for **3c**
**Figure S12.** ^13^C NMR spectrum for **3c**
**Figure S13.** IR spectrum for **3d**
**Figure S14.** ^1^H NMR spectrum for **3d**
**Figure S15.** ^13^C NMR spectrum for **3d**
**Figure S16.** IR spectrum for **3e**
**Figure S17.** ^1^H NMR spectrum for **3e**
**Figure S18.** ^13^C NMR spectrum for **3e**
**Figure S19.** IR spectrum for **3f**
**Figure S20.** ^1^H NMR spectrum for **3f**
**Figure S21.** ^13^C NMR spectrum for **3f**
**Figure S22.** IR spectrum for **3g**
**Figure S23.** ^1^H NMR spectrum for **3g**
**Figure S24.** ^13^C NMR spectrum for **3g**
**Figure S25.** IR spectrum for **3h**
**Figure S26.** ^1^H NMR spectrum for **3h**
**Figure S27.** ^13^C NMR spectrum for **3h**
**Figure S28.** IR spectrum for **3i**
**Figure S29.** ^1^H NMR spectrum for **3i**
**Figure S30.** ^13^C NMR spectrum for **3i**
**Figure S31.** IR spectrum for **3j**
**Figure S32.** ^1^H NMR spectrum for **3j**
**Figure S33.** ^13^C NMR spectrum for **3j**
**Figure S34.** IR spectrum for **3k**
**Figure S35.** ^1^H NMR spectrum for **3k**
**Figure S36.** ^13^C NMR spectrum for **3k**
**Figure S37.** IR spectrum for **3l**
**Figure S38.** ^1^H NMR spectrum for **3l**
**Figure S39.** ^13^C NMR spectrum for **3l**
**Figure S40.** IR spectrum for **3m**
**Figure S41.** ^1^H NMR spectrum for **3m**
**Figure S42.** ^13^C NMR spectrum for **3m**
**Figure S43.** IR spectrum for **3n**
**Figure S44.** ^1^H NMR spectrum for **3n**
**Figure S45.** ^13^C NMR spectrum for **3n**
**Figure S46.** IR spectrum for **3o**
**Figure S47.** ^1^H NMR spectrum for **3o**
**Figure S48.** ^13^C NMR spectrum for **3o**
**Figure S49.** IR spectrum for **3p**
**Figure S50.** ^1^H NMR spectrum for **3p**
**Figure S51.** ^13^C NMR spectrum for **3p**
**Figure S52.** IR spectrum for **3q**
**Figure S53.** ^1^H NMR spectrum for **3q**
**Figure S54.** ^13^C NMR spectrum for **3q**
**Figure S55.** IR spectrum for **3r**
**Figure S56.** ^1^H NMR spectrum for **3r**
**Figure S57.** ^13^C NMR spectrum for **3r**
**Figure S58.** IR spectrum for **3s**
**Figure S59.** ^1^H NMR spectrum for **3s**
**Figure S60.** ^13^C NMR spectrum for **3s**
**Figure S61.** IR spectrum for **3t**
**Figure S62.** ^1^H NMR spectrum for **3t**
**Figure S63.** ^13^C NMR spectrum for **3t**

**Figure S1.** IR spectrum for **2**

**Figure S2.** ^1^H NMR spectrum for **2**

**Figure S3.** ^13^C NMR spectrum for **2**

**Figure S4.** IR spectrum for **3a**

**Figure S5.** ^1^H NMR spectrum for **3a**

**Figure S6.** ^13^C NMR spectrum for **3a**

**Figure S7.** IR spectrum for **3b**

**Figure S8.** ^1^H NMR spectrum for **3b**

**Figure S9.** ^13^C NMR spectrum for **3b**

**Figure S10.** IR spectrum for **3c**

**Figure S11.** ^1^H NMR spectrum for **3c**

**Figure S12.** ^13^C NMR spectrum for **3c**

**Figure S13.** IR spectrum for **3d**

**Figure S14.** ^1^H NMR spectrum for **3d**

**Figure S15.** ^13^C NMR spectrum for **3d**

**Figure S16.** IR spectrum for **3e**

**Figure S17.** ^1^H NMR spectrum for **3e**

**Figure S18.** ^13^C NMR spectrum for **3e**

**Figure S19.** IR spectrum for **3f**

**Figure S20.** ^1^H NMR spectrum for **3f**

**Figure S21.** ^13^C NMR spectrum for **3f**

**Figure S22.** IR spectrum for **3g**

**Figure S23.** ^1^H NMR spectrum for **3g**

**Figure S24.** ^13^C NMR spectrum for **3g**

**Figure S25.** IR spectrum for **3h**

**Figure S26.** ^1^H NMR spectrum for **3h**

**Figure S27.** ^13^C NMR spectrum for **3h**

**Figure S28.** IR spectrum for **3i**

**Figure S29.** ^1^H NMR spectrum for **3i**

**Figure S30.** ^13^C NMR spectrum for **3i**

**Figure S31.** IR spectrum for **3j**

**Figure S32.** ^1^H NMR spectrum for **3j**

**Figure S33.** ^13^C NMR spectrum for **3j**

**Figure S34.** IR spectrum for **3k**

**Figure S35.** ^1^H NMR spectrum for **3k**

**Figure S36.** ^13^C NMR spectrum for **3k**

**Figure S37.** IR spectrum for **3l**

**Figure S38.** ^1^H NMR spectrum for **3l**

**Figure S39.** ^13^C NMR spectrum for **3l**

**Figure S40.** IR spectrum for **3m**

**Figure S41.** ^1^H NMR spectrum for **3m**

**Figure S42.** ^13^C NMR spectrum for **3m**

**Figure S43.** IR spectrum for **3n**

**Figure S44.** ^1^H NMR spectrum for **3n**

**Figure S45.** ^13^C NMR spectrum for **3n**

**Figure S46.** IR spectrum for **3o**

**Figure S47.** ^1^H NMR spectrum for **3o**

**Figure S48.** ^13^C NMR spectrum for **3o**

**Figure S49.** IR spectrum for **3p**

**Figure S50.** ^1^H NMR spectrum for **3p**

**Figure S51.** ^13^C NMR spectrum for **3p**

**Figure S52.** IR spectrum for **3q**

**Figure S53.** ^1^H NMR spectrum for **3q**

**Figure S54.** ^13^C NMR spectrum for **3q**

**Figure S55.** IR spectrum for **3r**

**Figure S56.** ^1^H NMR spectrum for **3r**

**Figure S57.** ^13^C NMR spectrum for **3r**

**Figure S58.** IR spectrum for **3s**

**Figure S59.** ^1^H NMR spectrum for **3s**

**Figure S60.** ^13^C NMR spectrum for **3s**

**Figure S61.** IR spectrum for **3t**

**Figure S62.** ^1^H NMR spectrum for **3t**

**Figure S63.** ^13^C NMR spectrum for **3t**
